# Supplementary figures and images for: Prescribing of anti-dementia medications in primary care: A retrospective cohort study in 1489 English General Practices
Source: PLoS One. 2026 Jun 1;21(6):e0347921. doi: 10.1371/journal.pone.0347921 (PMC13225638; doi:10.1371/journal.pone.0347921)

### Supplementary figure 1: Description of the cohort study design

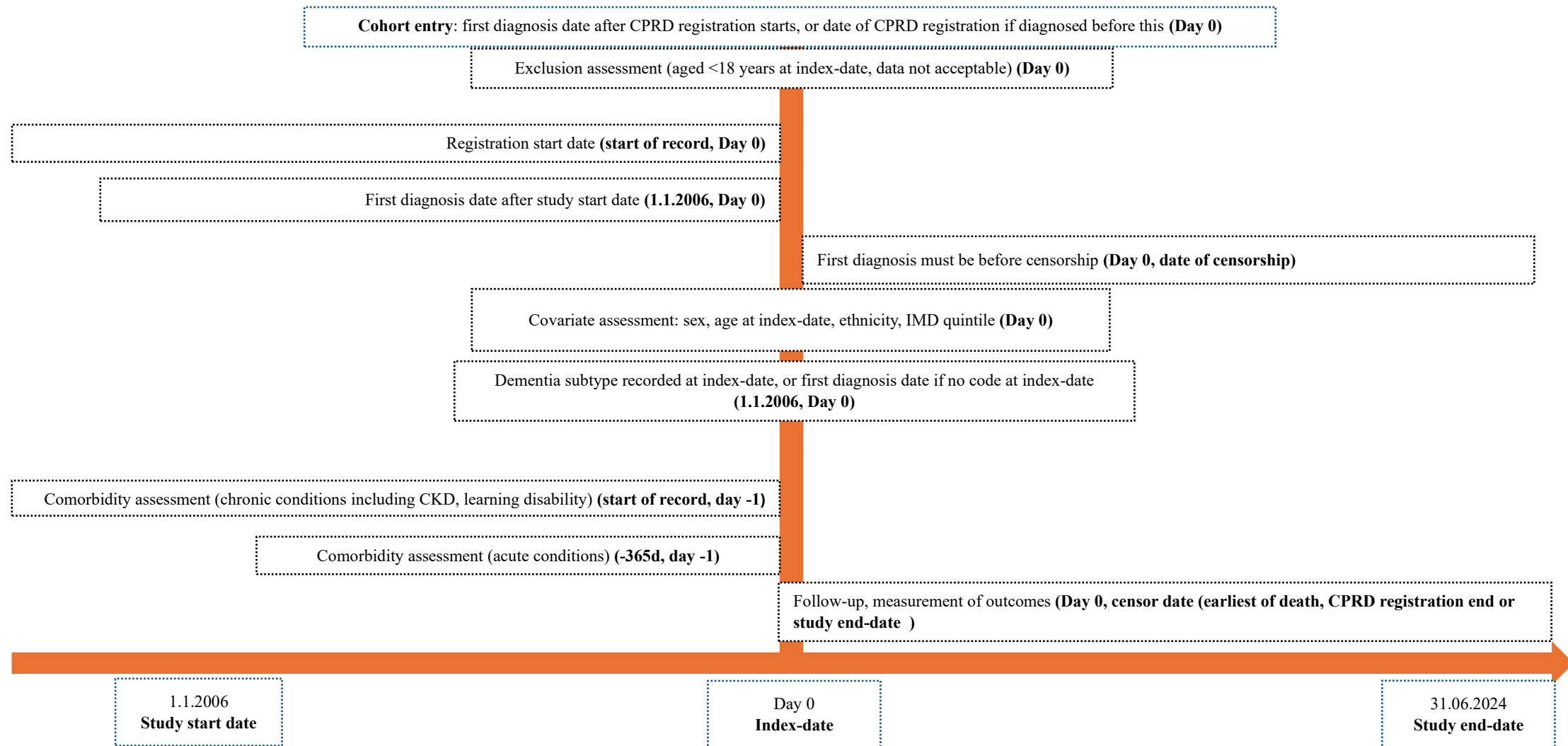

Supplement: S1 Fig — (PDF) [file pone.0347921.s001.pdf]
