# Supplementary material for: Prescribing of anti-dementia medications in primary care: A retrospective cohort study in 1489 English General Practices
Source: PLoS One. 2026 Jun 1;21(6):e0347921. doi: 10.1371/journal.pone.0347921 (PMC13225638; doi:10.1371/journal.pone.0347921)
Supplement: S2 Fig — (PDF) [file pone.0347921.s002.pdf]

Supplementary figure 2a: Criteria for incident co-prescribing (AChE-inhibitor and memantine)

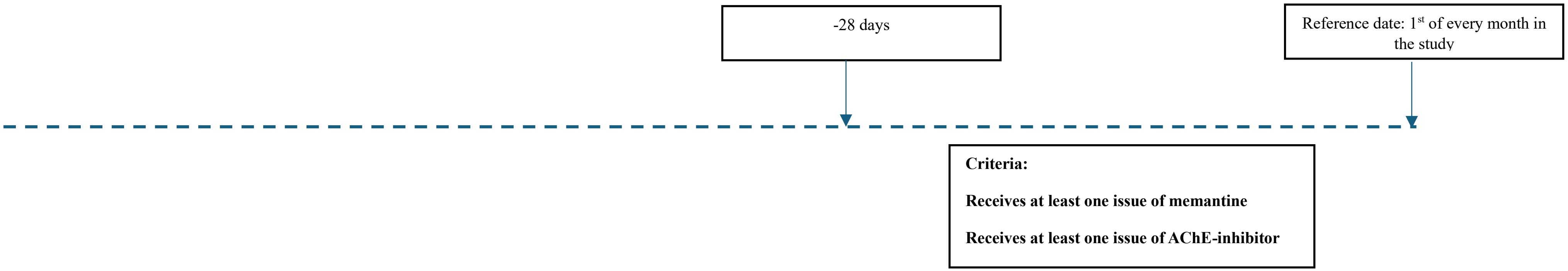

Supplementary figure 2b: Criteria for incident switching (AChE-inhibitor to memantine)

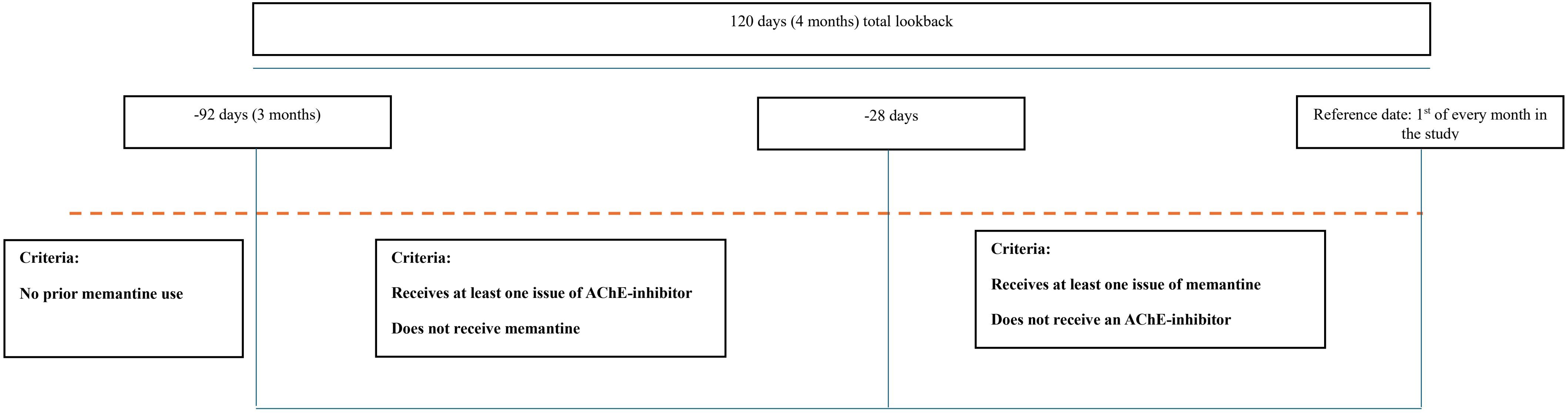

Supplementary figure 2c: Criteria for prevalent switch (AChE-inhibitor to memantine)

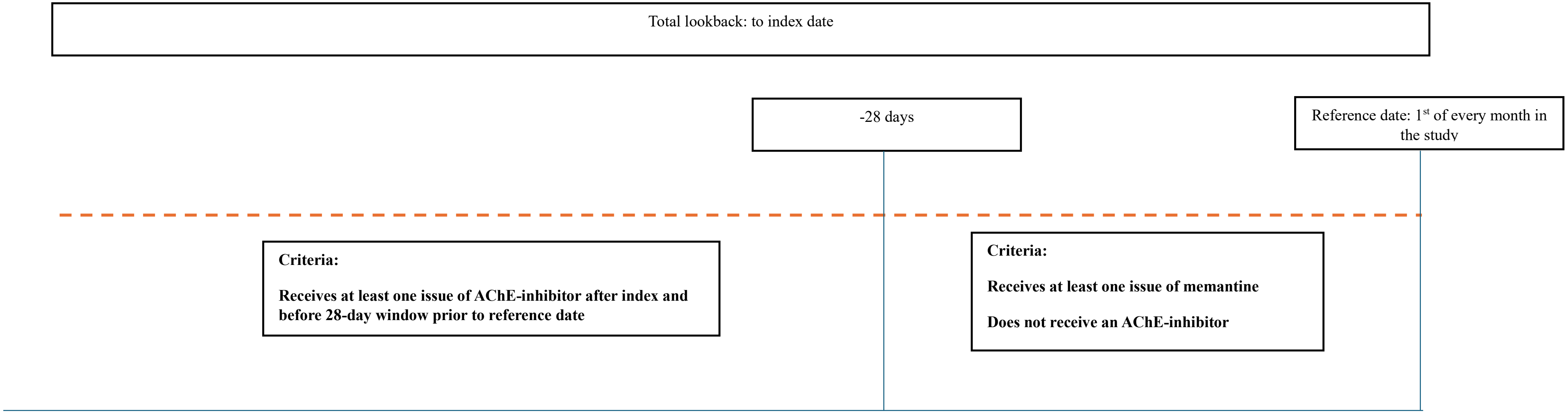
Total lookback: to index date
-28 days
Reference date: 1st of every month in the study
Criteria:
Receives at least one issue of AChE-inhibitor after index and before 28-day window prior to reference date
Criteria:
Receives at least one issue of memantine
Does not receive an AChE-inhibitor
