# Supplementary material for: Prescribing of anti-dementia medications in primary care: A retrospective cohort study in 1489 English General Practices
Source: PLoS One. 2026 Jun 1;21(6):e0347921. doi: 10.1371/journal.pone.0347921 (PMC13225638; doi:10.1371/journal.pone.0347921)
Supplement: S1 Table — (PDF) [file pone.0347921.s003.pdf]

**Supplementary figure 3a: Sensitivity analysis examining people ever issued acetyl-cholinesterase inhibitors, including people with unspecified dementia, AD, LBD and mixed (AD/LBD) subtypes. (n=425,332)**

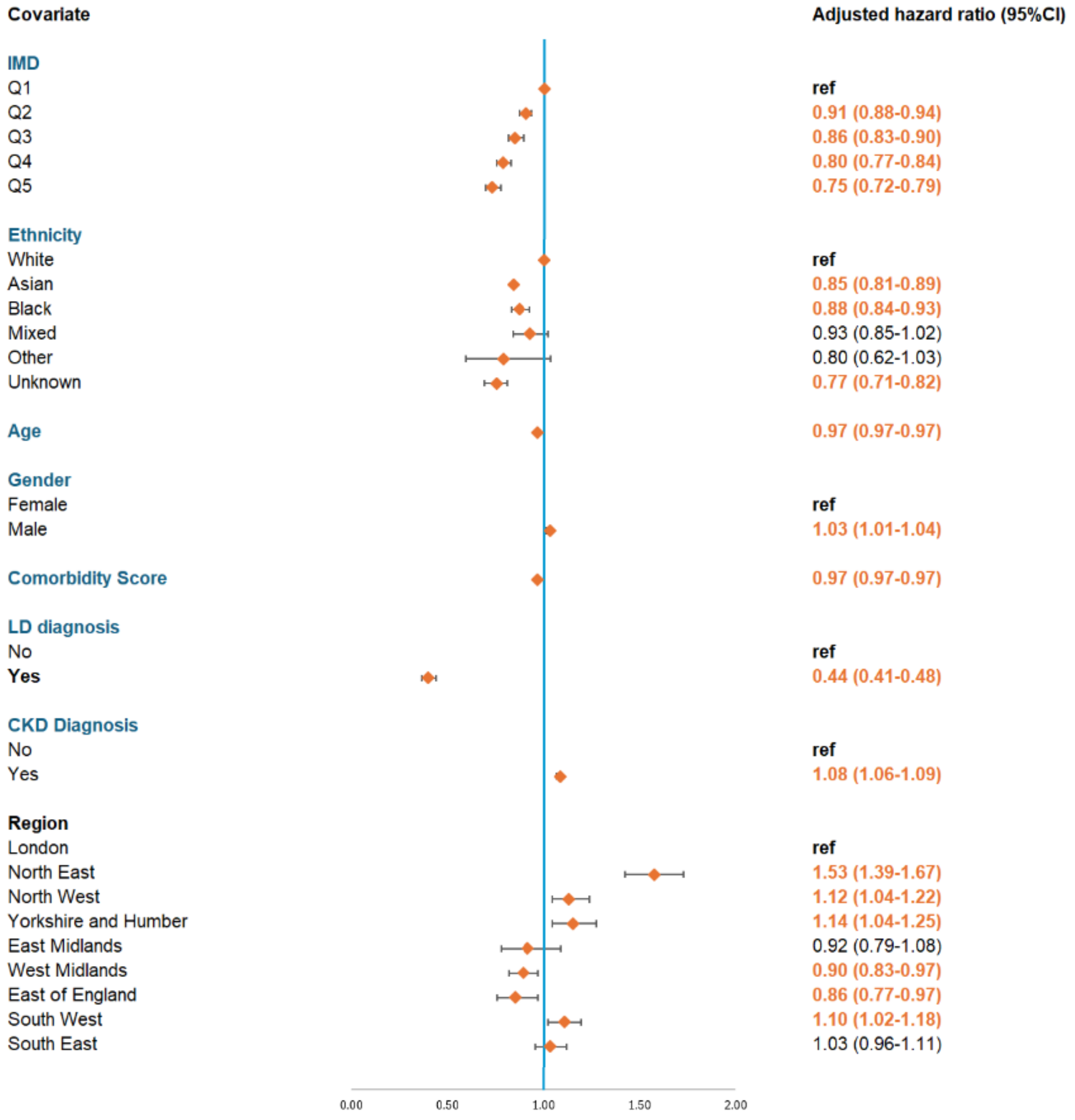


Footnote: Quintile 1 is least deprived, quintile 5 is most deprived.

**Supplementary figure 3b: Sensitivity analysis examining people ever issued memantine including people with unspecified dementia, AD, LBD and mixed (AD/LBD) subtypes. (n=425,332)**

| Covariate | Adjusted hazard ratio (95%CI) |
|---|---|
| **IMD** | |
| Q1 | **ref** |
| Q2 | 0.97 (0.93-1.02) |
| Q3 | 0.95 (0.90-1.00) |
| Q4 | 0.90 (0.85-0.95) |
| Q5 | 0.88 (0.82-0.93) |
| **Ethnicity** | |
| White | **ref** |
| Asian | 0.87 (0.82-0.93) |
| Black | 0.79 (0.74-0.85) |
| Mixed | 0.85 (0.74-0.97) |
| Other | 1.08 (0.82-1.43) |
| Unknown | 0.79 (0.67-0.92) |
| **Age** | 0.99 (0.99-0.99) |
| **Gender** | |
| Female | **ref** |
| Male | 1.25 (1.23-1.27) |
| **Comorbidity Score** | 1.00 (0.99-1.00) |
| **LD diagnosis** | |
| No | **ref** |
| **Yes** | 0.50 (0.44-0.57) |
| **CKD Diagnosis** | |
| No | **ref** |
| Yes | 0.94 (0.91-0.96) |
| **Region** | |
| London | **ref** |
| North East | 1.30 (1.15-1.48) |
| North West | 1.21 (1.09-1.33) |
| Yorkshire and Humber | 0.92 (0.78-1.09) |
| East Midlands | 0.65 (0.56-0.75) |
| West Midlands | 0.70(0.62-0.79) |
| East of England | 0.54 (0.46-0.65) |
| South West | 0.95 (0.87-1.05) |
| South East | 0.94 (0.85-1.04) |

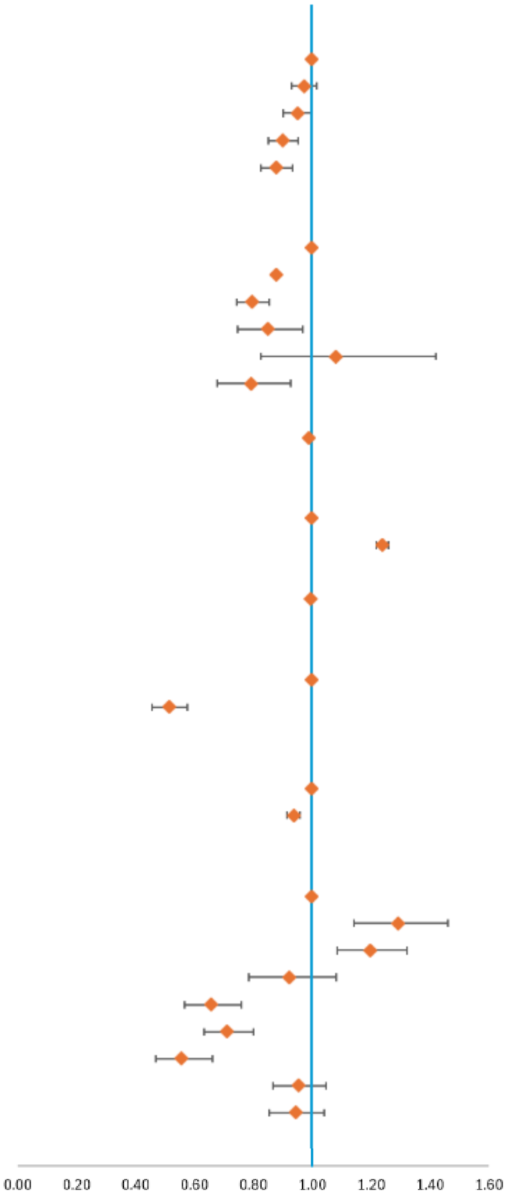


Footnote: Quintile 1 is least deprived, quintile 5 is most deprived.
