# Supplementary material for: Prescribing of anti-dementia medications in primary care: A retrospective cohort study in 1489 English General Practices
Source: PLoS One. 2026 Jun 1;21(6):e0347921. doi: 10.1371/journal.pone.0347921 (PMC13225638; doi:10.1371/journal.pone.0347921)

Supplementary figure 4a: Percentage of patients prescribed acetyl-cholinesterase inhibitors for each practice by patient-level deprivation quintile (violin plots[1]) (n=1489 practices)

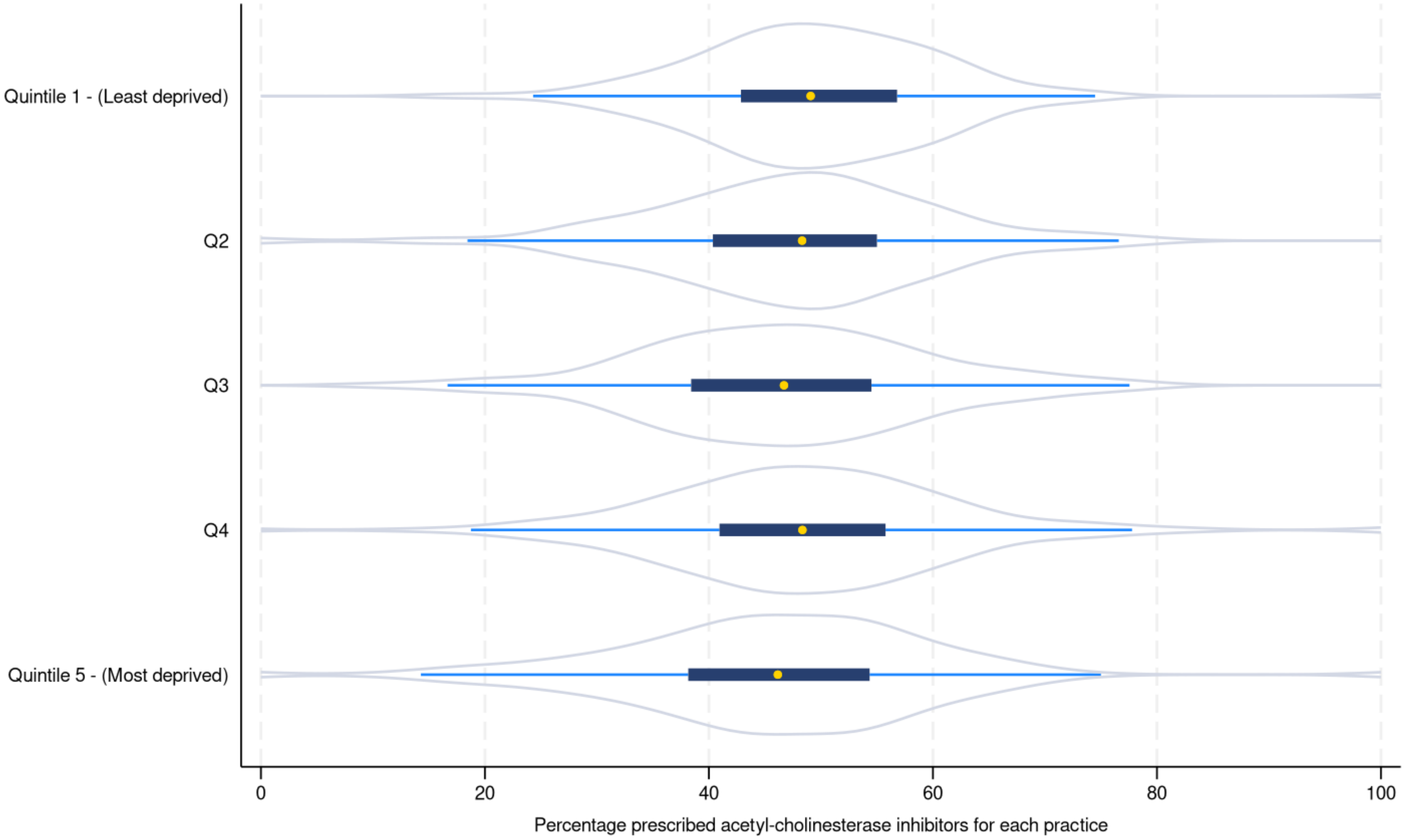

[1] A violin plot visualizes the distribution of data by combining the features of a box plot with a density plo t. The "violin" shape represents the data's density; the wider the section, the more data points are concentrated at that value. The inner dot marks the median, and the mini box and whiskers shows the interquartile range and data spread.

41. Hintze JL, Nelson RD. Violin Plots: A Box Plot-Density Trace Synergism. *The American Statistician*. 1998;52(2):181-4.

Supplementary figure 4b: Percentage of patients prescribed memantine for each practice by patient-level deprivation quintile (violin plots) (n=1489 practices)

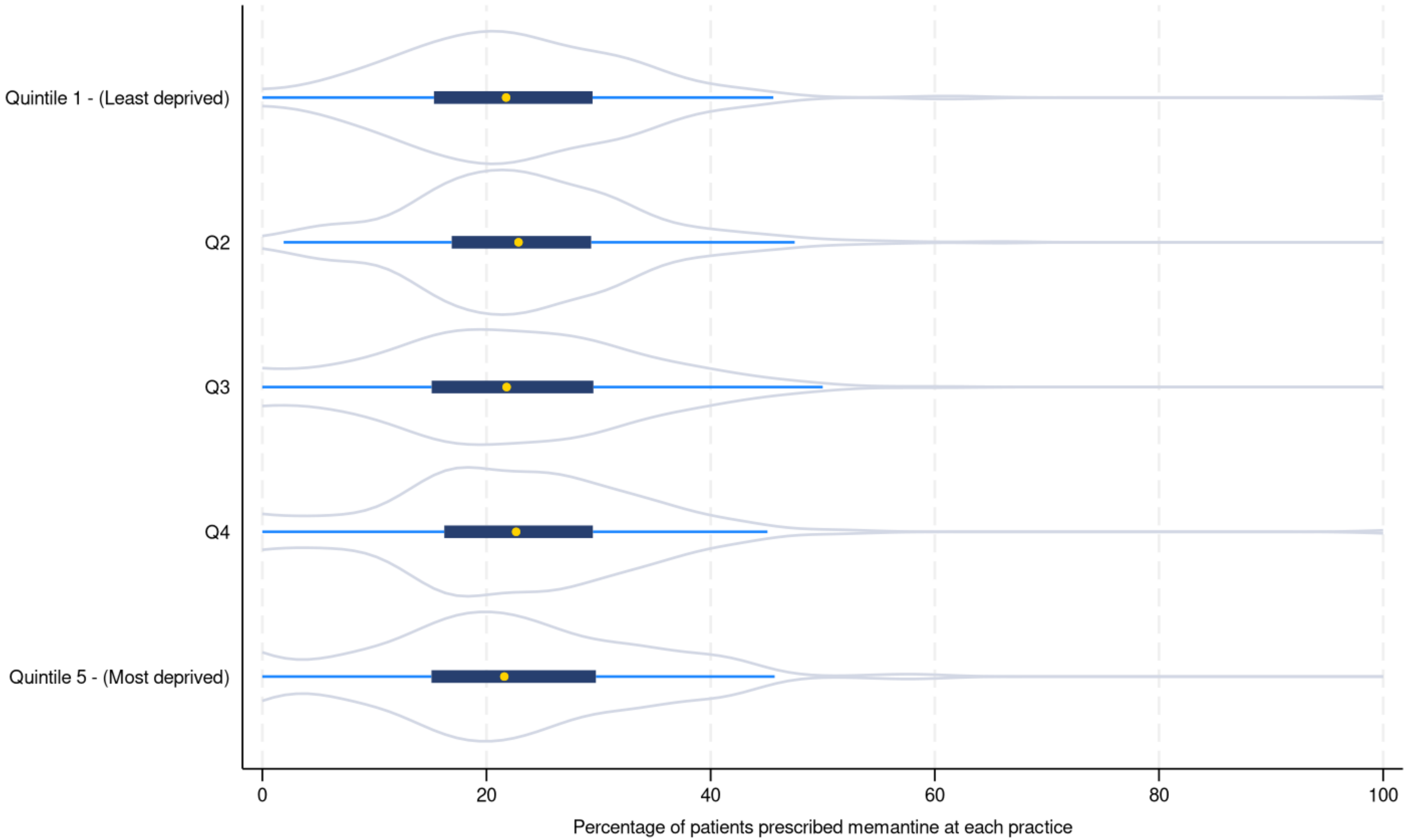

Supplement: S2 Table — (PDF) [file pone.0347921.s004.pdf]
