# Supplementary material for: Prescribing of anti-dementia medications in primary care: A retrospective cohort study in 1489 English General Practices
Source: PLoS One. 2026 Jun 1;21(6):e0347921. doi: 10.1371/journal.pone.0347921 (PMC13225638; doi:10.1371/journal.pone.0347921)

Supplementary figure 5a: Competing risks model for ever issued acetyl-cholinesterase inhibitor (n=24,252) [run on random 10% sample]

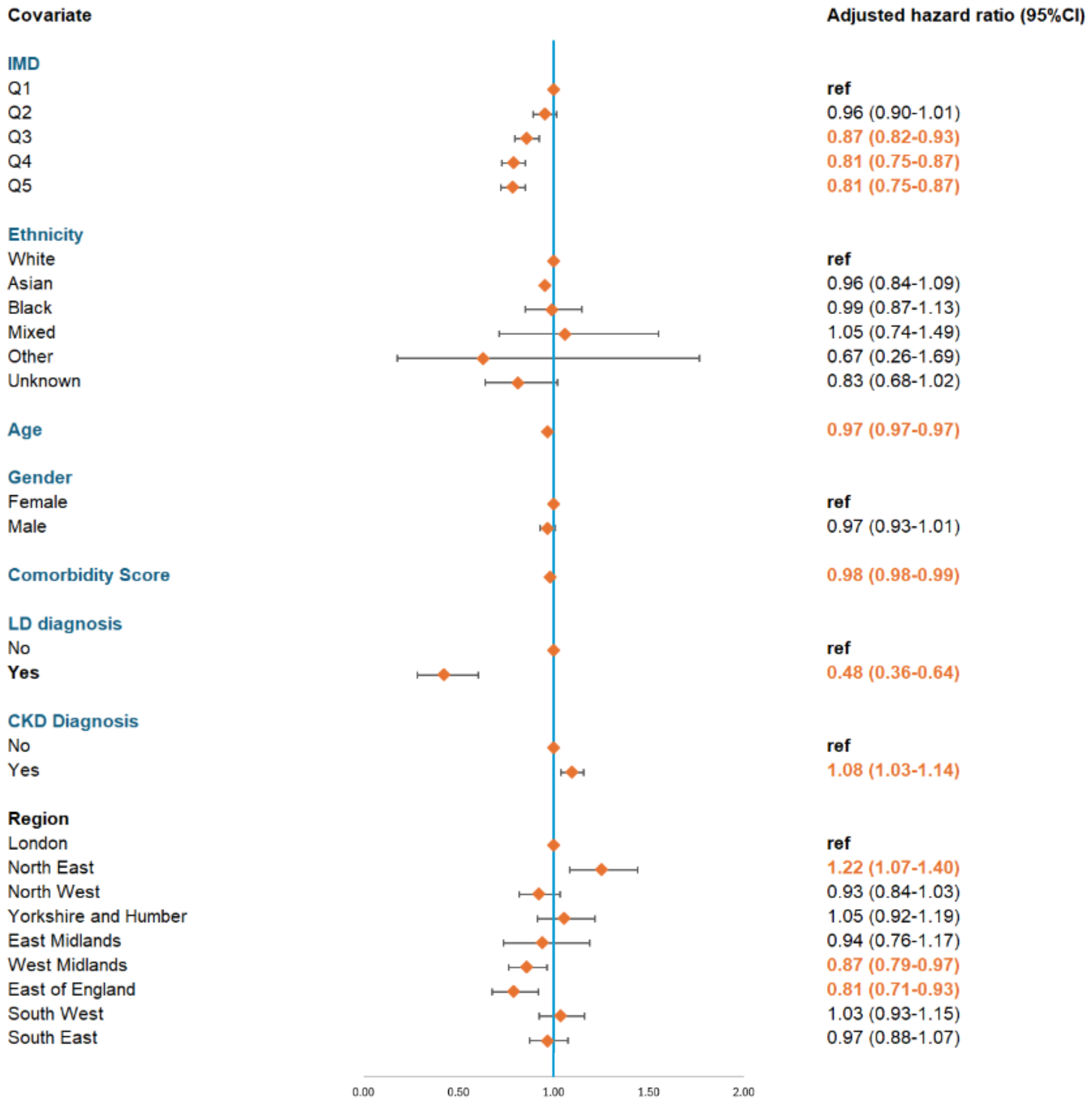

Supplementary figure 5b: Competing risks model for ever issued memantine (n=24,252) [run on random 10% sample]

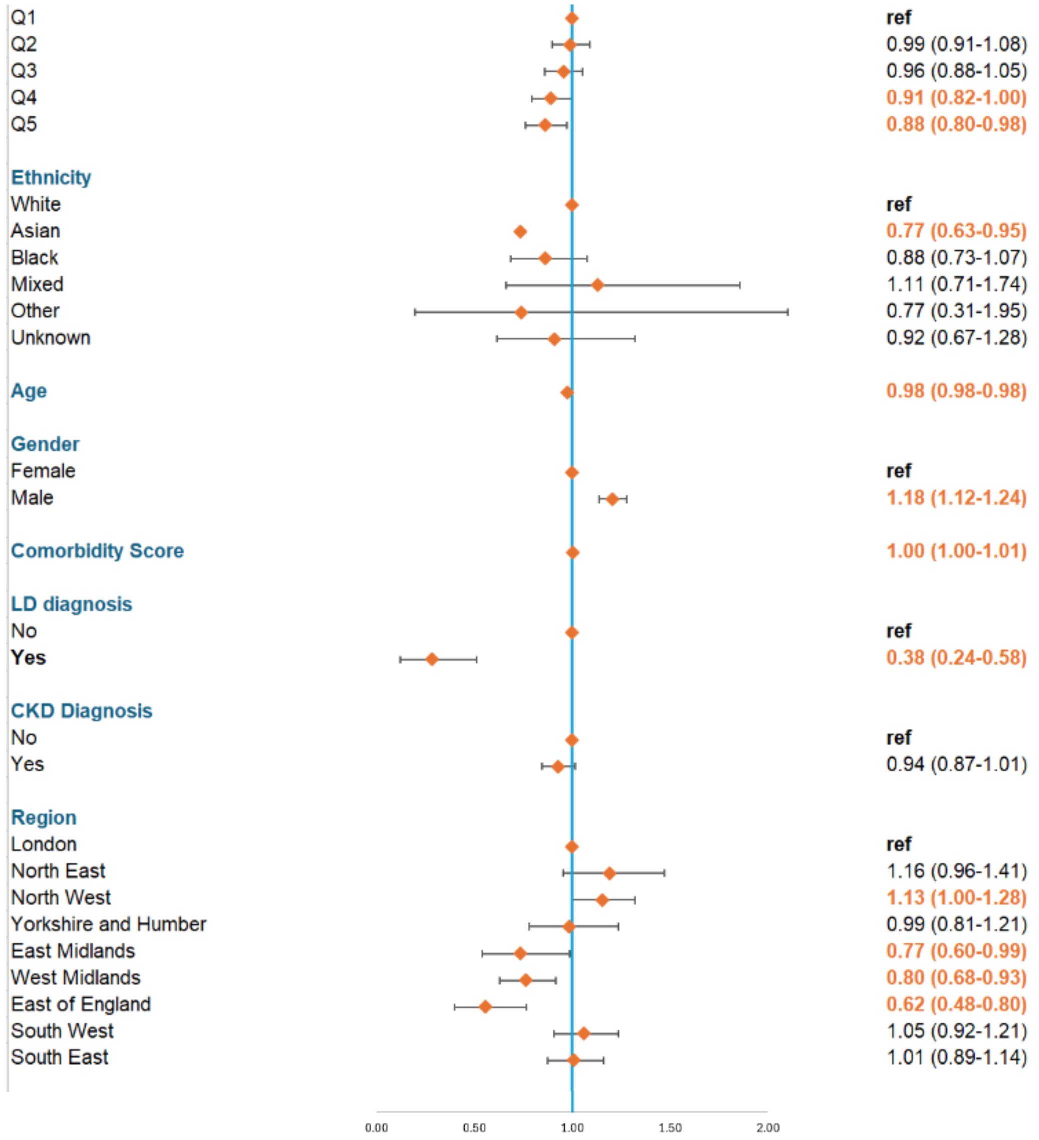

Supplement: S3 Fig — (n = 425,332); Sensitivity analysis examining people ever issued memantine including people with unspecified dementia, AD, LBD and mixed (AD/LBD) subtypes. (n = 425,332). (PDF) [file pone.0347921.s005.pdf]
