# Supplementary material for: Prescribing of anti-dementia medications in primary care: A retrospective cohort study in 1489 English General Practices
Source: PLoS One. 2026 Jun 1;21(6):e0347921. doi: 10.1371/journal.pone.0347921 (PMC13225638; doi:10.1371/journal.pone.0347921)

Supplementary figure 6a: Parametric regression model: Adjusted hazard ratio for likelihood of ever receiving an acetyl-cholinesterase inhibitor based on patient characteristics (n=242,007)

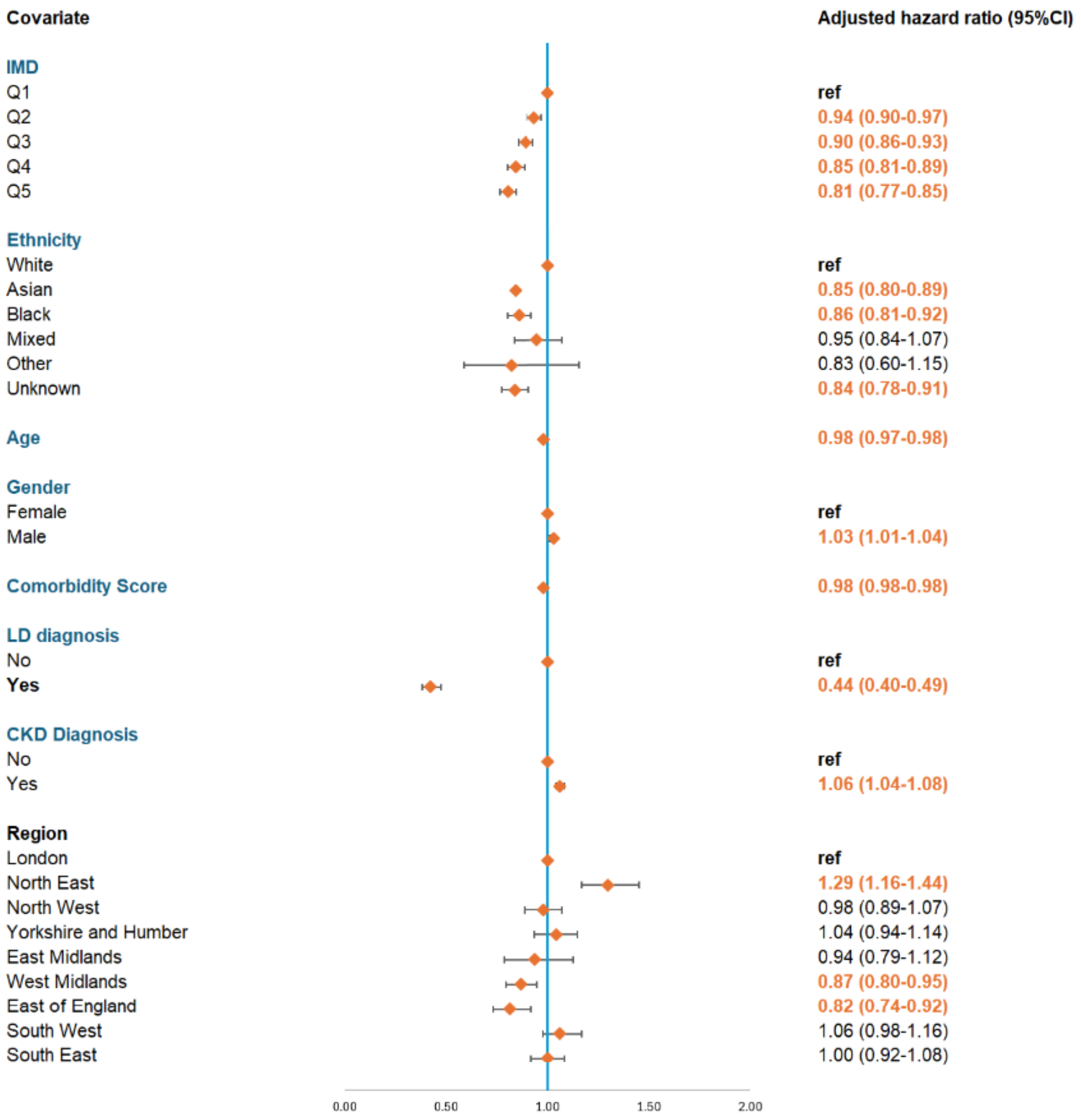

Supplementary figure 6b: Parametric regression model: Adjusted hazard ratio for likelihood of ever receiving memantine based on patient characteristics (n=242,007)

Covariate	Adjusted hazard ratio (95%CI)
**IMD**	
Q1	ref
Q2	1.00 (0.95-1.04)
Q3	0.99 (0.94-1.04)
Q4	0.97 (0.91-1.03)
Q5	0.96 (0.90-1.02)
**Ethnicity**	
White	ref
Asian	0.89 (0.83-0.96)
Black	0.79 (0.73-0.85)
Mixed	0.85 (0.73-1.00)
Other	1.09 (0.79-1.51)
Unknown	0.90 (0.77-1.06)
**Age**	0.99 (0.99-0.99)
**Gender**	
Female	ref
Male	1.23 (1.20-1.25)
**Comorbidity Score**	1.00 (1.00-1.01)
**LD diagnosis**	
No	ref
**Yes**	0.58 (0.50-0.67)
**CKD Diagnosis**	
No	ref
Yes	0.91 (0.88-0.93)
**Region**	
London	ref
North East	1.19 (1.05-1.34)
North West	1.17 (1.06-1.29)
Yorkshire and Humber	0.94 (0.80-1.09)
East Midlands	0.72 (0.63-0.83)
West Midlands	0.78 (0.70-0.88)
East of England	0.58 (0.49-0.69)
South West	1.00 (0.91-1.10)
South East	0.98 (0.89-1.08)

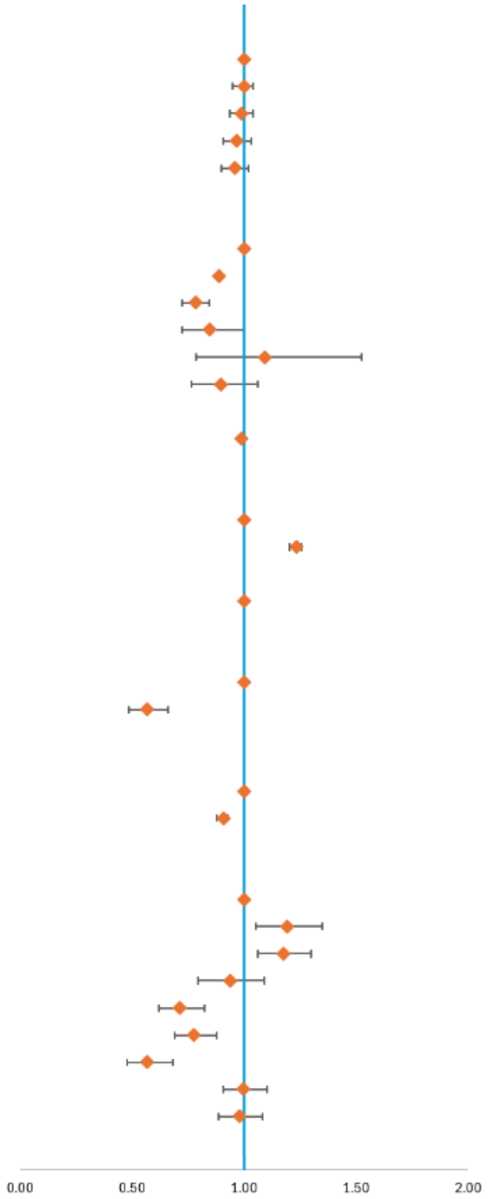

Supplement: S4 Fig — (PDF) [file pone.0347921.s006.pdf]
