# Supplementary material for: Prescribing of anti-dementia medications in primary care: A retrospective cohort study in 1489 English General Practices
Source: PLoS One. 2026 Jun 1;21(6):e0347921. doi: 10.1371/journal.pone.0347921 (PMC13225638; doi:10.1371/journal.pone.0347921)

Supplementary figure 7a: Adjusted hazard ratio for likelihood of ever receiving acetyl-cholinesterase inhibitors based on patient characteristics within 24 months of index date, (Cox regression) (n=242,007)

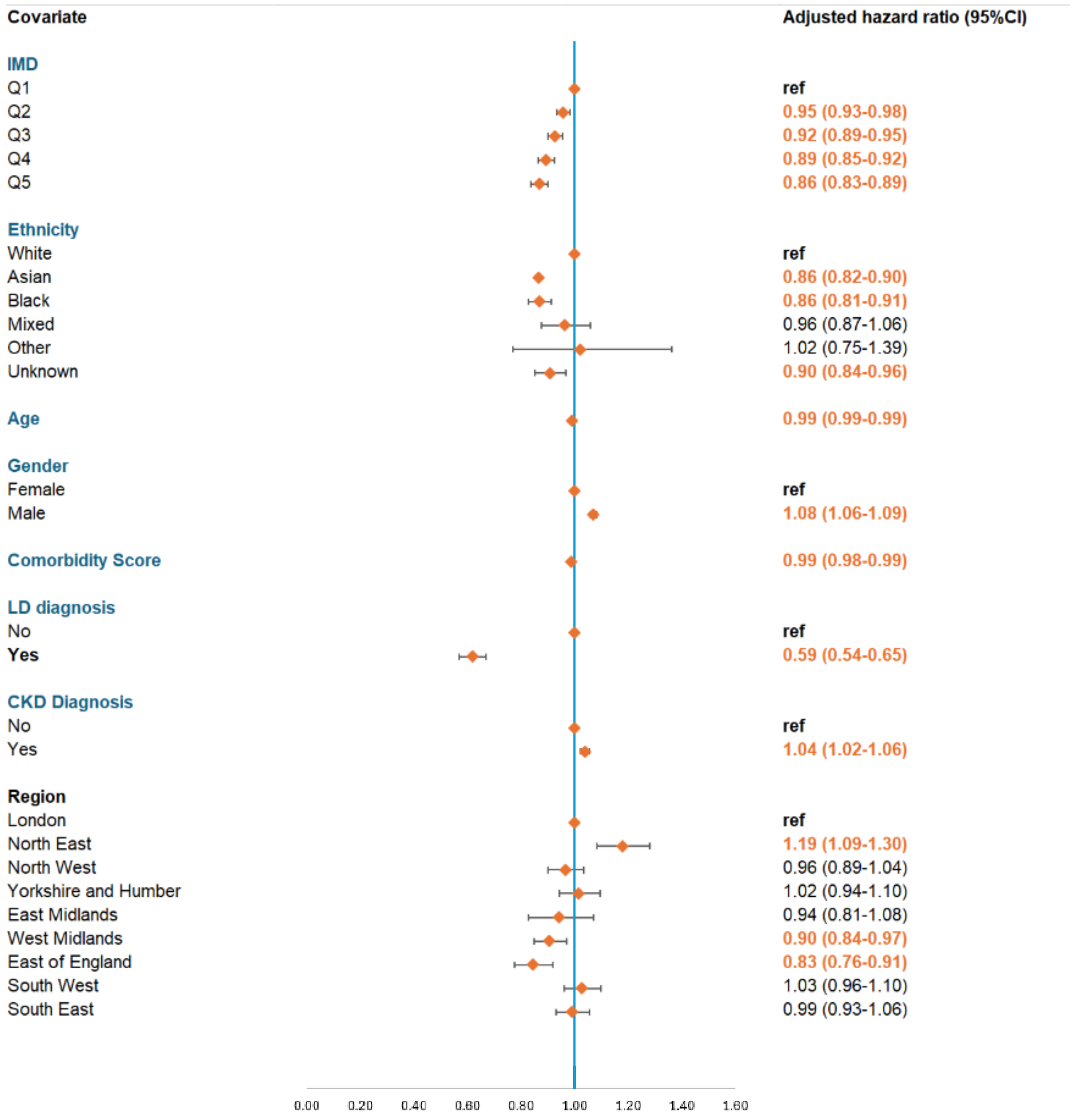

Supplementary figure 7b: Adjusted hazard ratio for likelihood of ever receiving memantine based on patient characteristics within 24 months of index date, (Cox regression) (n=242,007)

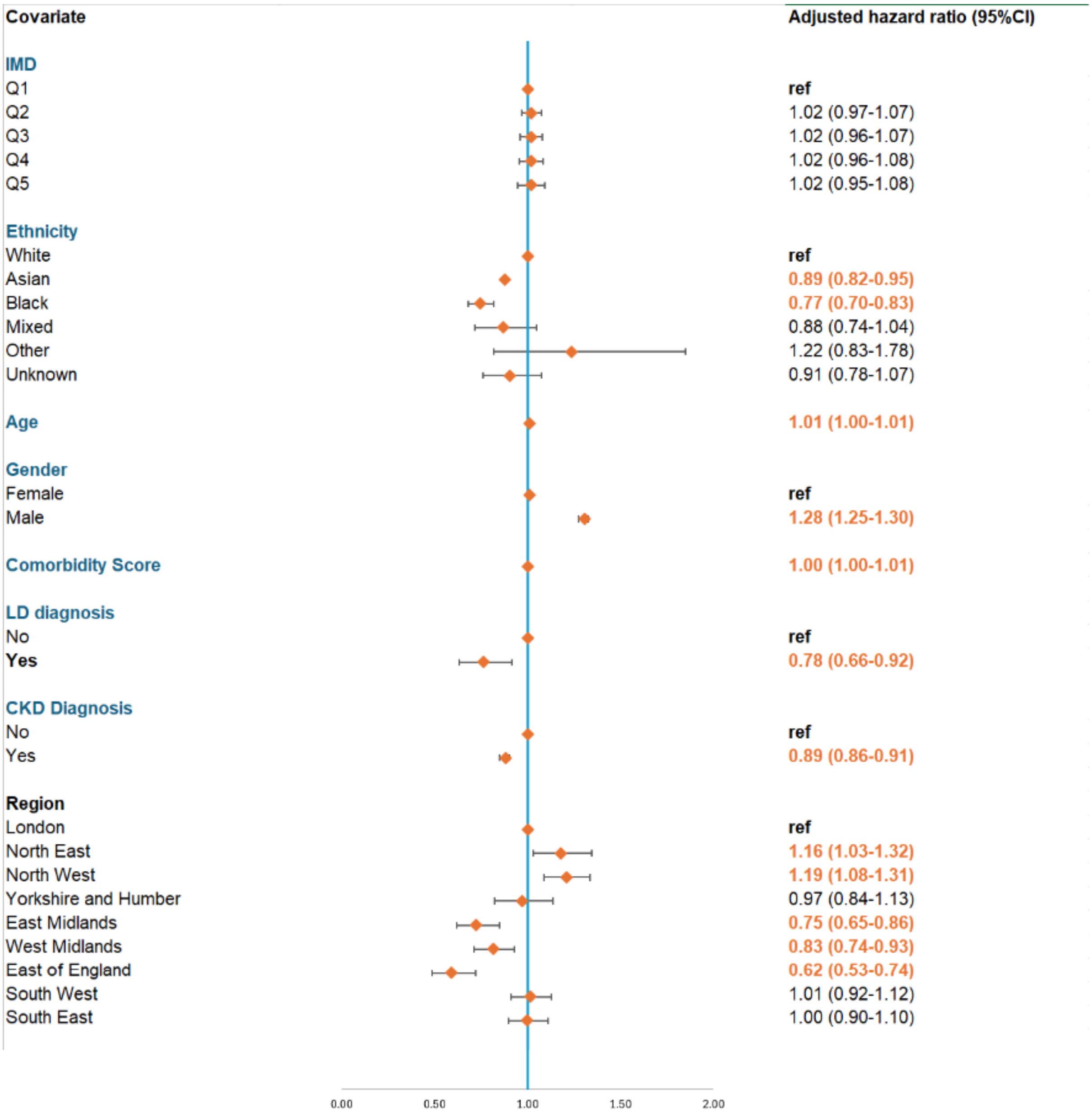

Supplement: S5 Fig — (PDF) [file pone.0347921.s007.pdf]
