# Supplementary material for: Prescribing of anti-dementia medications in primary care: A retrospective cohort study in 1489 English General Practices
Source: PLoS One. 2026 Jun 1;21(6):e0347921. doi: 10.1371/journal.pone.0347921 (PMC13225638; doi:10.1371/journal.pone.0347921)
Supplement: S6 Fig — (PDF) [file pone.0347921.s008.pdf]

<u>Supplementary table 1a: Number of people ever receiving anti-dementia medications, based on year of diagnosis</u>

	Year of diagnosis																			
	**2006**	**2007**	**2008**	**2009**	**2010**	**2011**	**2012**	**2013**	**2014**	**2015**	**2016**	**2017**	**2018**	**2019**	**2020**	**2021**	**2022**	**2023**	**2024**	**Total**
Number ever receiving memantine	384	355	484	698	1,033	1,486	2,211	2,784	3,508	4,027	4,578	5,332	5,850	6,461	5,851	6,184	3,431	2,923	742	58,322
Percentage ever receiving memantine	6.0	6	7.3	9.1	12.1	15.9	19.1	20.9	22.2	24.0	25.7	27.4	28.5	30.6	32.9	33.2	32.3	28.5	17.8	24.1
Number ever receiving AChE-I	2,576	2,545	3,068	3,848	4,271	5,145	6,372	7,410	8,304	8,344	8,629	8,993	9,256	9,431	7,384	7,968	4,917	4,697	1,340	114,498
Percentage ever receiving AChE-I	40.8	43.0	46.3	50.0	51.5	55.1	55.0	55.7	52.5	49.8	48.5	46.3	45.1	44.7	41.5	42.7	46.3	45.7	32.1	47.3
**Total diagnosed in each year**	**6,319**	**5,914**	**6,622**	**7,701**	**8,294**	**9,338**	**11,586**	**13,310**	**15,832**	**16,769**	**17,795**	**19,438**	**20,516**	**21,081**	**17,792**	**18,642**	**10,619**	**10,269**	**4,170**	**242,007**

Supplementary Table 1b: Number of people receiving the first prescription of AChEIs and memantine each year, as a percentage of the total number in the study each year.

	Year of study																			
	**2006**	**2007**	**2008**	**2009**	**2010**	**2011**	**2012**	**2013**	**2014**	**2015**	**2016**	**2017**	**2018**	**2019**	**2020**	**2021**	**2022**	**2023**	**2024**	**Total**
Number initiated on memantine	115 (2.2%)	98 (1.0%)	128 (0.9%)	171 (0.9%)	247 (1.1%)	714 (2.7%)	1,621 (4.9%)	2258 (5.8%)	3122 (6.6%)	3752 (7.0%)	4535 (7.7%)	5101 (7.9%)	5823 (8.5%)	6594 (9.1%)	6464 (9.0%)	6906 (10.0%)	4525 (7.4%)	4345 (7.7%)	1803 (3.8%)	58322
Number initiated on AChEIs (%)	1471 (27.6%)	2016 (20.6%)	2443 (17.5%)	3328 (18.2%)	3852 (17.2%)	4708 (17.5%)	6045 (18.4%)	7740 (19.8%)	8440 (18.0%)	8878 (16.5%)	8923 (15.1%)	9150 (14.2%)	9332 (13.6%)	9650 (13.3%)	7806 (10.9%)	8163 (11.8%)	5288 (8.6%)	5193 (9.2%)	2072 (4.4%)	114498
**Total in the study in each year***	**5330**	**775**	**13934**	**18327**	**22403**	**26885**	**32881**	**39165**	**47012**	**53885**	**59237**	**64367**	**68807**	**72438**	**71884**	**69353**	**61463**	**56437**	**47637**	**N=242007†**

***includes people in the study for at least one day in each year**
†Some people are counted in more than one year so this is not a row total
