# Supplementary material for: Prescribing of anti-dementia medications in primary care: A retrospective cohort study in 1489 English General Practices
Source: PLoS One. 2026 Jun 1;21(6):e0347921. doi: 10.1371/journal.pone.0347921 (PMC13225638; doi:10.1371/journal.pone.0347921)
Supplement: S3 Table — (PDF) [file pone.0347921.s009.pdf]

Supplementary Table 2a: Trends in prescribing of both memantine and AChEIs over the study period			
**Month of study reference date**	**Number receiving memantine in preceding 28 days to reference date**	**Number in the study on the reference date**	**Percentage receiving both medications in preceding 28 days to reference date**
01-Jan-06	0	424	0.00
01-Feb-06	0	785	0.00
01-Mar-06	<5	1191	<0.5
01-Apr-06	<5	1507	<0.5
01-May-06	6	1961	0.31
01-Jun-06	6	2428	0.25
01-Jul-06	7	2827	0.25
01-Aug-06	8	3231	0.25
01-Sep-06	10	3587	0.28
01-Oct-06	8	4058	0.20
01-Nov-06	14	4397	0.32
01-Dec-06	14	4707	0.30
01-Jan-07	10	5073	0.20
01-Feb-07	16	5324	0.30
01-Mar-07	13	5649	0.23
01-Apr-07	17	5903	0.29
01-May-07	21	6191	0.34
01-Jun-07	18	6458	0.28
01-Jul-07	18	6732	0.27
01-Aug-07	18	7022	0.26
01-Sep-07	23	7256	0.32
01-Oct-07	26	7618	0.34
01-Nov-07	29	7883	0.37
01-Dec-07	26	8048	0.32
01-Jan-08	22	8398	0.26
01-Feb-08	23	8681	0.26
01-Mar-08	22	8918	0.25
01-Apr-08	18	9175	0.20
01-May-08	25	9376	0.27
01-Jun-08	22	9622	0.23
01-Jul-08	24	9910	0.24
01-Aug-08	25	10166	0.25
01-Sep-08	23	10466	0.22
01-Oct-08	28	10835	0.26
01-Nov-08	23	11104	0.21
01-Dec-08	23	11371	0.20
01-Jan-09	28	11702	0.24
01-Feb-09	30	11933	0.25
01-Mar-09	30	12299	0.24
01-Apr-09	30	12623	0.24
01-May-09	32	12875	0.25
01-Jun-09	33	13267	0.25
01-Jul-09	30	13590	0.22

01-Aug-09	36	13794	0.26
01-Sep-09	37	14102	0.26
01-Oct-09	41	14418	0.28
01-Nov-09	41	14695	0.28
01-Dec-09	46	14964	0.31
01-Jan-10	37	15149	0.24
01-Feb-10	39	15348	0.25
01-Mar-10	41	15652	0.26
01-Apr-10	44	15873	0.28
01-May-10	48	16098	0.30
01-Jun-10	46	16454	0.28
01-Jul-10	55	16725	0.33
01-Aug-10	45	16900	0.27
01-Sep-10	50	17235	0.29
01-Oct-10	54	17576	0.31
01-Nov-10	53	17949	0.30
01-Dec-10	55	18138	0.30
01-Jan-11	52	18414	0.28
01-Feb-11	60	18713	0.32
01-Mar-11	64	19189	0.33
01-Apr-11	65	19302	0.34
01-May-11	63	19636	0.32
01-Jun-11	77	19972	0.39
01-Jul-11	83	20271	0.41
01-Aug-11	97	20535	0.47
01-Sep-11	96	20805	0.46
01-Oct-11	106	21101	0.50
01-Nov-11	115	21502	0.53
01-Dec-11	118	21774	0.54
01-Jan-12	144	22202	0.65
01-Feb-12	163	22604	0.72
01-Mar-12	169	23005	0.73
01-Apr-12	195	23277	0.84
01-May-12	195	23749	0.82
01-Jun-12	209	23976	0.87
01-Jul-12	215	24420	0.88
01-Aug-12	231	24823	0.93
01-Sep-12	258	25220	1.02
01-Oct-12	247	25789	0.96
01-Nov-12	247	26148	0.94
01-Dec-12	272	26375	1.03
01-Jan-13	266	26899	0.99
01-Feb-13	298	27279	1.09
01-Mar-13	303	27563	1.10
01-Apr-13	314	27823	1.13
01-May-13	311	28243	1.10

01-Jun-13	306	28715	1.07
01-Jul-13	310	29347	1.06
01-Aug-13	337	29753	1.13
01-Sep-13	336	30180	1.11
01-Oct-13	366	30778	1.19
01-Nov-13	361	31218	1.16
01-Dec-13	373	31514	1.18
01-Jan-14	380	31938	1.19
01-Feb-14	386	32208	1.20
01-Mar-14	408	32669	1.25
01-Apr-14	431	33005	1.31
01-May-14	446	33463	1.33
01-Jun-14	480	33884	1.42
01-Jul-14	475	34500	1.38
01-Aug-14	501	34915	1.43
01-Sep-14	510	35519	1.44
01-Oct-14	538	36289	1.48
01-Nov-14	515	37118	1.39
01-Dec-14	531	37966	1.40
01-Jan-15	518	38550	1.34
01-Feb-15	558	38644	1.44
01-Mar-15	558	39276	1.42
01-Apr-15	562	39547	1.42
01-May-15	556	39725	1.40
01-Jun-15	574	40166	1.43
01-Jul-15	595	40714	1.46
01-Aug-15	615	40966	1.50
01-Sep-15	622	41382	1.50
01-Oct-15	652	41813	1.56
01-Nov-15	647	42153	1.53
01-Dec-15	653	42348	1.54
01-Jan-16	645	42523	1.52
01-Feb-16	680	42813	1.59
01-Mar-16	683	43259	1.58
01-Apr-16	683	43404	1.57
01-May-16	691	43666	1.58
01-Jun-16	718	44131	1.63
01-Jul-16	728	44435	1.64
01-Aug-16	745	44951	1.66
01-Sep-16	721	45454	1.59
01-Oct-16	762	45769	1.66
01-Nov-16	769	46180	1.67
01-Dec-16	783	46277	1.69
01-Jan-17	786	46468	1.69
01-Feb-17	811	46629	1.74
01-Mar-17	816	46995	1.74

01-Apr-17	824	47043	1.75
01-May-17	862	47470	1.82
01-Jun-17	855	47848	1.79
01-Jul-17	888	48288	1.84
01-Aug-17	891	48815	1.83
01-Sep-17	891	49012	1.82
01-Oct-17	914	49535	1.85
01-Nov-17	945	49888	1.89
01-Dec-17	951	49828	1.91
01-Jan-18	946	49906	1.90
01-Feb-18	958	49668	1.93
01-Mar-18	969	49654	1.95
01-Apr-18	986	49583	1.99
01-May-18	1041	49935	2.08
01-Jun-18	992	50243	1.97
01-Jul-18	1013	50820	1.99
01-Aug-18	1024	51164	2.00
01-Sep-18	1039	51474	2.02
01-Oct-18	1085	52147	2.08
01-Nov-18	1125	52509	2.14
01-Dec-18	1159	52689	2.20
01-Jan-19	1169	53162	2.20
01-Feb-19	1253	53329	2.35
01-Mar-19	1290	53566	2.41
01-Apr-19	1278	53739	2.38
01-May-19	1308	53984	2.42
01-Jun-19	1375	54236	2.54
01-Jul-19	1385	54756	2.53
01-Aug-19	1462	55014	2.66
01-Sep-19	1452	55228	2.63
01-Oct-19	1497	55653	2.69
01-Nov-19	1515	55792	2.72
01-Dec-19	1558	55660	2.80
01-Jan-20	1553	55818	2.78
01-Feb-20	1640	55729	2.94
01-Mar-20	1641	55832	2.94
01-Apr-20	1690	55305	3.06
01-May-20	1620	53061	3.05
01-Jun-20	1603	52555	3.05
01-Jul-20	1628	52556	3.10
01-Aug-20	1677	52300	3.21
01-Sep-20	1609	52487	3.07
01-Oct-20	1724	52676	3.27
01-Nov-20	1744	52452	3.32
01-Dec-20	1744	52232	3.34
01-Jan-21	1790	51831	3.45

01-Feb-21	1840	51012	3.61
01-Mar-21	1839	51185	3.59
01-Apr-21	1867	51283	3.64
01-May-21	1893	51523	3.67
01-Jun-21	1888	51802	3.64
01-Jul-21	1939	51980	3.73
01-Aug-21	1964	51914	3.78
01-Sep-21	1924	51895	3.71
01-Oct-21	2046	52003	3.93
01-Nov-21	2007	52084	3.85
01-Dec-21	2056	52030	3.95
01-Jan-22	2054	51346	4.00
01-Feb-22	2121	50863	4.17
01-Mar-22	2063	50500	4.09
01-Apr-22	2013	49802	4.04
01-May-22	1941	49409	3.93
01-Jun-22	1987	49015	4.05
01-Jul-22	1919	48725	3.94
01-Aug-22	1931	48387	3.99
01-Sep-22	1851	47945	3.86
01-Oct-22	1881	47702	3.94
01-Nov-22	1878	47486	3.95
01-Dec-22	1837	47106	3.90
01-Jan-23	1787	46569	3.84
01-Feb-23	1802	45906	3.93
01-Mar-23	1747	45747	3.82
01-Apr-23	1774	45345	3.91
01-May-23	1764	45174	3.90
01-Jun-23	1727	45035	3.83
01-Jul-23	1745	44932	3.88
01-Aug-23	1735	44865	3.87
01-Sep-23	1708	44769	3.82
01-Oct-23	1704	44764	3.81
01-Nov-23	1725	44641	3.86
01-Dec-23	1691	44286	3.82
01-Jan-24	1688	44071	3.83
01-Feb-24	1681	43713	3.85
01-Mar-24	1685	43491	3.87
01-Apr-24	1657	43305	3.83
01-May-24	1710	43219	3.96
01-Jun-24	1669	42579	3.92

Supplementary table 2b: Incidence of switching from AChEIs to memantine in preceding 28 days			
**Month of Study**	**Number switching in each month (incidence of switch)**	**Number in the study on reference date**	**Percentage**
01-Jan-06	0	424	0.00
01-Feb-06	0	785	0.00
01-Mar-06	0	1191	0.00
01-Apr-06	0	1507	0.00
01-May-06	0	1961	0.00
01-Jun-06	0	2428	0.00
01-Jul-06	0	2827	0.00
01-Aug-06	0	3231	0.00
01-Sep-06	<5	3587	<0.05
01-Oct-06	<5	4058	<0.05
01-Nov-06	0	4397	0.00
01-Dec-06	<5	4707	<0.05
01-Jan-07	0	5073	0.00
01-Feb-07	0	5324	0.00
01-Mar-07	<5	5649	<0.05
01-Apr-07	0	5903	0.00
01-May-07	0	6191	0.00
01-Jun-07	0	6458	0.00
01-Jul-07	<5	6732	<0.05
01-Aug-07	0	7022	0.00
01-Sep-07	0	7256	0.00
01-Oct-07	0	7618	0.00
01-Nov-07	0	7883	0.00
01-Dec-07	0	8048	0.00
01-Jan-08	<5	8398	<0.05
01-Feb-08	0	8681	0.00
01-Mar-08	<5	8918	<0.05
01-Apr-08	0	9175	0.00
01-May-08	0	9376	0.00
01-Jun-08	<5	9622	<0.05
01-Jul-08	<5	9910	<0.05
01-Aug-08	<5	10166	<0.05
01-Sep-08	0	10466	0.00
01-Oct-08	<5	10835	<0.05
01-Nov-08	<5	11104	<0.05
01-Dec-08	<5	11371	<0.05
01-Jan-09	<5	11702	<0.05
01-Feb-09	0	11933	0.00
01-Mar-09	0	12299	0.00
01-Apr-09	<5	12623	<0.05
01-May-09	<5	12875	<0.05
01-Jun-09	<5	13267	<0.05
01-Jul-09	<5	13590	<0.05
01-Aug-09	0	13794	0.00

01-Sep-09	<5	14102	<0.05
01-Oct-09	0	14418	0.00
01-Nov-09	<5	14695	<0.05
01-Dec-09	0	14964	0.00
01-Jan-10	0	15149	0.00
01-Feb-10	<5	15348	<0.05
01-Mar-10	<5	15652	<0.05
01-Apr-10	<5	15873	<0.05
01-May-10	<5	16098	<0.05
01-Jun-10	0	16454	0.00
01-Jul-10	<5	16725	<0.05
01-Aug-10	<5	16900	<0.05
01-Sep-10	<5	17235	<0.05
01-Oct-10	0	17576	0.00
01-Nov-10	<5	17949	<0.05
01-Dec-10	8	18138	0.04
01-Jan-11	0	18414	0.00
01-Feb-11	<5	18713	<0.05
01-Mar-11	<5	19189	<0.05
01-Apr-11	<5	19302	<0.05
01-May-11	<5	19636	<0.05
01-Jun-11	5	19972	0.03
01-Jul-11	<5	20271	<0.05
01-Aug-11	<5	20535	<0.05
01-Sep-11	11	20805	0.05
01-Oct-11	<5	21101	0.02
01-Nov-11	10	21502	<0.05
01-Dec-11	10	21774	0.05
01-Jan-12	10	22202	0.05
01-Feb-12	15	22604	0.07
01-Mar-12	15	23005	0.07
01-Apr-12	13	23277	0.06
01-May-12	6	23749	0.03
01-Jun-12	11	23976	0.05
01-Jul-12	17	24420	0.07
01-Aug-12	9	24823	0.04
01-Sep-12	11	25220	0.04
01-Oct-12	9	25789	0.03
01-Nov-12	14	26148	0.05
01-Dec-12	10	26375	0.04
01-Jan-13	18	26899	0.07
01-Feb-13	9	27279	0.03
01-Mar-13	16	27563	0.06
01-Apr-13	16	27823	0.06
01-May-13	10	28243	0.04
01-Jun-13	10	28715	0.03

01-Jul-13	17	29347	0.06
01-Aug-13	22	29753	0.07
01-Sep-13	21	30180	0.07
01-Oct-13	21	30778	0.07
01-Nov-13	22	31218	0.07
01-Dec-13	19	31514	0.06
01-Jan-14	19	31938	0.06
01-Feb-14	23	32208	0.07
01-Mar-14	24	32669	0.07
01-Apr-14	16	33005	0.05
01-May-14	26	33463	0.08
01-Jun-14	15	33884	0.04
01-Jul-14	16	34500	0.05
01-Aug-14	29	34915	0.08
01-Sep-14	23	35519	0.06
01-Oct-14	24	36289	0.07
01-Nov-14	26	37118	0.07
01-Dec-14	24	37966	0.06
01-Jan-15	25	38550	0.06
01-Feb-15	36	38644	0.09
01-Mar-15	23	39276	0.06
01-Apr-15	24	39547	0.06
01-May-15	28	39725	0.07
01-Jun-15	16	40166	0.04
01-Jul-15	33	40714	0.08
01-Aug-15	23	40966	0.06
01-Sep-15	25	41382	0.06
01-Oct-15	24	41813	0.06
01-Nov-15	27	42153	0.06
01-Dec-15	25	42348	0.06
01-Jan-16	28	42523	0.07
01-Feb-16	36	42813	0.08
01-Mar-16	37	43259	0.09
01-Apr-16	26	43404	0.06
01-May-16	30	43666	0.07
01-Jun-16	34	44131	0.08
01-Jul-16	29	44435	0.07
01-Aug-16	29	44951	0.06
01-Sep-16	34	45454	0.07
01-Oct-16	33	45769	0.07
01-Nov-16	32	46180	0.07
01-Dec-16	33	46277	0.07
01-Jan-17	36	46468	0.08
01-Feb-17	36	46629	0.08
01-Mar-17	40	46995	0.09
01-Apr-17	31	47043	0.07

01-May-17	30	47470	0.06
01-Jun-17	33	47848	0.07
01-Jul-17	40	48288	0.08
01-Aug-17	28	48815	0.06
01-Sep-17	42	49012	0.09
01-Oct-17	28	49535	0.06
01-Nov-17	30	49888	0.06
01-Dec-17	24	49828	0.05
01-Jan-18	29	49906	0.06
01-Feb-18	41	49668	0.08
01-Mar-18	31	49654	0.06
01-Apr-18	24	49583	0.05
01-May-18	41	49935	0.08
01-Jun-18	33	50243	0.07
01-Jul-18	32	50820	0.06
01-Aug-18	33	51164	0.06
01-Sep-18	35	51474	0.07
01-Oct-18	37	52147	0.07
01-Nov-18	46	52509	0.09
01-Dec-18	34	52689	0.06
01-Jan-19	42	53162	0.08
01-Feb-19	34	53329	0.06
01-Mar-19	31	53566	0.06
01-Apr-19	41	53739	0.08
01-May-19	38	53984	0.07
01-Jun-19	39	54236	0.07
01-Jul-19	35	54756	0.06
01-Aug-19	35	55014	0.06
01-Sep-19	39	55228	0.07
01-Oct-19	35	55653	0.06
01-Nov-19	27	55792	0.05
01-Dec-19	32	55660	0.06
01-Jan-20	41	55818	0.07
01-Feb-20	32	55729	0.06
01-Mar-20	44	55832	0.08
01-Apr-20	44	55305	0.08
01-May-20	36	53061	0.07
01-Jun-20	36	52555	0.07
01-Jul-20	39	52556	0.07
01-Aug-20	21	52300	0.04
01-Sep-20	31	52487	0.06
01-Oct-20	24	52676	0.05
01-Nov-20	28	52452	0.05
01-Dec-20	35	52232	0.07
01-Jan-21	30	51831	0.06
01-Feb-21	41	51012	0.08

01-Mar-21	38	51185	0.07
01-Apr-21	39	51283	0.08
01-May-21	42	51523	0.08
01-Jun-21	36	51802	0.07
01-Jul-21	39	51980	0.08
01-Aug-21	17	51914	0.03
01-Sep-21	21	51895	0.04
01-Oct-21	34	52003	0.07
01-Nov-21	43	52084	0.08
01-Dec-21	35	52030	0.07
01-Jan-22	36	51346	0.07
01-Feb-22	34	50863	0.07
01-Mar-22	33	50500	0.07
01-Apr-22	31	49802	0.06
01-May-22	23	49409	0.05
01-Jun-22	27	49015	0.06
01-Jul-22	32	48725	0.07
01-Aug-22	31	48387	0.06
01-Sep-22	32	47945	0.07
01-Oct-22	24	47702	0.05
01-Nov-22	33	47486	0.07
01-Dec-22	24	47106	0.05
01-Jan-23	44	46569	0.09
01-Feb-23	32	45906	0.07
01-Mar-23	42	45747	0.09
01-Apr-23	41	45345	0.09
01-May-23	33	45174	0.07
01-Jun-23	37	45035	0.08
01-Jul-23	42	44932	0.09
01-Aug-23	29	44865	0.06
01-Sep-23	38	44769	0.08
01-Oct-23	26	44764	0.06
01-Nov-23	39	44641	0.09
01-Dec-23	38	44286	0.09
01-Jan-24	41	44071	0.09
01-Feb-24	52	43713	0.12
01-Mar-24	28	43491	0.06
01-Apr-24	33	43305	0.08
01-May-24	29	43219	0.07
01-Jun-24	41	42579	0.10

Supplementary table 2c: Prevalence of switching from AChEIs to memantine			
Month of study reference date	Number who have switched and are still received memantine and no ACHE-I in 28 days prior to reference date (Prevalence)	Number in the study on reference date	Percentage
01-Jan-06	0	424	0.00
01-Feb-06	0	785	0.00
01-Mar-06	0	1191	0.00
01-Apr-06	0	1507	0.00
01-May-06	<5	1961	<0.05
01-Jun-06	0	2428	0.00
01-Jul-06	<5	2827	<0.05
01-Aug-06	0	3231	0.00
01-Sep-06	<5	3587	<0.05
01-Oct-06	<5	4058	<0.05
01-Nov-06	<5	4397	<0.05
01-Dec-06	<5	4707	<0.05
01-Jan-07	<5	5073	<0.05
01-Feb-07	<5	5324	<0.05
01-Mar-07	<5	5649	<0.05
01-Apr-07	<5	5903	<0.05
01-May-07	<5	6191	<0.05
01-Jun-07	<5	6458	<0.05
01-Jul-07	5	6732	0.07
01-Aug-07	<5	7022	<0.06
01-Sep-07	7	7256	0.10
01-Oct-07	7	7618	0.09
01-Nov-07	6	7883	0.08
01-Dec-07	8	8048	0.10
01-Jan-08	10	8398	0.12
01-Feb-08	9	8681	0.10
01-Mar-08	12	8918	0.13
01-Apr-08	13	9175	0.14
01-May-08	10	9376	0.11
01-Jun-08	12	9622	0.12
01-Jul-08	16	9910	0.16
01-Aug-08	18	10166	0.18
01-Sep-08	15	10466	0.14
01-Oct-08	18	10835	0.17
01-Nov-08	19	11104	0.17
01-Dec-08	19	11371	0.17
01-Jan-09	22	11702	0.19
01-Feb-09	17	11933	0.14
01-Mar-09	19	12299	0.15
01-Apr-09	18	12623	0.14
01-May-09	22	12875	0.17
01-Jun-09	20	13267	0.15
01-Jul-09	21	13590	0.15
01-Aug-09	15	13794	0.11

01-Sep-09	19	14102	0.13
01-Oct-09	18	14418	0.12
01-Nov-09	22	14695	0.15
01-Dec-09	18	14964	0.12
01-Jan-10	24	15149	0.16
01-Feb-10	21	15348	0.14
01-Mar-10	31	15652	0.20
01-Apr-10	20	15873	0.13
01-May-10	25	16098	0.16
01-Jun-10	25	16454	0.15
01-Jul-10	23	16725	0.14
01-Aug-10	36	16900	0.21
01-Sep-10	26	17235	0.15
01-Oct-10	31	17576	0.18
01-Nov-10	26	17949	0.14
01-Dec-10	42	18138	0.23
01-Jan-11	31	18414	0.17
01-Feb-11	41	18713	0.22
01-Mar-11	41	19189	0.21
01-Apr-11	38	19302	0.20
01-May-11	48	19636	0.24
01-Jun-11	52	19972	0.26
01-Jul-11	58	20271	0.29
01-Aug-11	58	20535	0.28
01-Sep-11	73	20805	0.35
01-Oct-11	86	21101	0.41
01-Nov-11	93	21502	0.43
01-Dec-11	111	21774	0.51
01-Jan-12	127	22202	0.57
01-Feb-12	154	22604	0.68
01-Mar-12	167	23005	0.73
01-Apr-12	185	23277	0.79
01-May-12	192	23749	0.81
01-Jun-12	213	23976	0.89
01-Jul-12	244	24420	1.00
01-Aug-12	235	24823	0.95
01-Sep-12	268	25220	1.06
01-Oct-12	273	25789	1.06
01-Nov-12	293	26148	1.12
01-Dec-12	306	26375	1.16
01-Jan-13	312	26899	1.16
01-Feb-13	326	27279	1.20
01-Mar-13	350	27563	1.27
01-Apr-13	366	27823	1.32
01-May-13	390	28243	1.38
01-Jun-13	375	28715	1.31

01-Jul-13	396	29347	1.35
01-Aug-13	414	29753	1.39
01-Sep-13	430	30180	1.42
01-Oct-13	459	30778	1.49
01-Nov-13	480	31218	1.54
01-Dec-13	478	31514	1.52
01-Jan-14	516	31938	1.62
01-Feb-14	547	32208	1.70
01-Mar-14	555	32669	1.70
01-Apr-14	558	33005	1.69
01-May-14	592	33463	1.77
01-Jun-14	600	33884	1.77
01-Jul-14	588	34500	1.70
01-Aug-14	632	34915	1.81
01-Sep-14	634	35519	1.78
01-Oct-14	666	36289	1.84
01-Nov-14	692	37118	1.86
01-Dec-14	703	37966	1.85
01-Jan-15	740	38550	1.92
01-Feb-15	778	38644	2.01
01-Mar-15	773	39276	1.97
01-Apr-15	799	39547	2.02
01-May-15	824	39725	2.07
01-Jun-15	799	40166	1.99
01-Jul-15	831	40714	2.04
01-Aug-15	859	40966	2.10
01-Sep-15	860	41382	2.08
01-Oct-15	890	41813	2.13
01-Nov-15	889	42153	2.11
01-Dec-15	943	42348	2.23
01-Jan-16	940	42523	2.21
01-Feb-16	978	42813	2.28
01-Mar-16	982	43259	2.27
01-Apr-16	987	43404	2.27
01-May-16	1044	43666	2.39
01-Jun-16	1051	44131	2.38
01-Jul-16	1049	44435	2.36
01-Aug-16	1069	44951	2.38
01-Sep-16	1098	45454	2.42
01-Oct-16	1158	45769	2.53
01-Nov-16	1147	46180	2.48
01-Dec-16	1211	46277	2.62
01-Jan-17	1213	46468	2.61
01-Feb-17	1232	46629	2.64
01-Mar-17	1214	46995	2.58
01-Apr-17	1274	47043	2.71

01-May-17	1239	47470	2.61
01-Jun-17	1255	47848	2.62
01-Jul-17	1277	48288	2.64
01-Aug-17	1321	48815	2.71
01-Sep-17	1316	49012	2.69
01-Oct-17	1366	49535	2.76
01-Nov-17	1331	49888	2.67
01-Dec-17	1363	49828	2.74
01-Jan-18	1362	49906	2.73
01-Feb-18	1359	49668	2.74
01-Mar-18	1359	49654	2.74
01-Apr-18	1330	49583	2.68
01-May-18	1409	49935	2.82
01-Jun-18	1340	50243	2.67
01-Jul-18	1367	50820	2.69
01-Aug-18	1374	51164	2.69
01-Sep-18	1388	51474	2.70
01-Oct-18	1425	52147	2.73
01-Nov-18	1425	52509	2.71
01-Dec-18	1467	52689	2.78
01-Jan-19	1459	53162	2.74
01-Feb-19	1492	53329	2.80
01-Mar-19	1441	53566	2.69
01-Apr-19	1473	53739	2.74
01-May-19	1484	53984	2.75
01-Jun-19	1481	54236	2.73
01-Jul-19	1529	54756	2.79
01-Aug-19	1477	55014	2.68
01-Sep-19	1511	55228	2.74
01-Oct-19	1548	55653	2.78
01-Nov-19	1567	55792	2.81
01-Dec-19	1581	55660	2.84
01-Jan-20	1574	55818	2.82
01-Feb-20	1572	55729	2.82
01-Mar-20	1582	55832	2.83
01-Apr-20	1635	55305	2.96
01-May-20	1559	53061	2.94
01-Jun-20	1516	52555	2.88
01-Jul-20	1539	52556	2.93
01-Aug-20	1543	52300	2.95
01-Sep-20	1477	52487	2.81
01-Oct-20	1498	52676	2.84
01-Nov-20	1506	52452	2.87
01-Dec-20	1500	52232	2.87
01-Jan-21	1486	51831	2.87
01-Feb-21	1469	51012	2.88

01-Mar-21	1483	51185	2.90
01-Apr-21	1525	51283	2.97
01-May-21	1547	51523	3.00
01-Jun-21	1525	51802	2.94
01-Jul-21	1534	51980	2.95
01-Aug-21	1504	51914	2.90
01-Sep-21	1485	51895	2.86
01-Oct-21	1542	52003	2.97
01-Nov-21	1545	52084	2.97
01-Dec-21	1539	52030	2.96
01-Jan-22	1517	51346	2.95
01-Feb-22	1566	50863	3.08
01-Mar-22	1503	50500	2.98
01-Apr-22	1505	49802	3.02
01-May-22	1437	49409	2.91
01-Jun-22	1454	49015	2.97
01-Jul-22	1406	48725	2.89
01-Aug-22	1429	48387	2.95
01-Sep-22	1400	47945	2.92
01-Oct-22	1412	47702	2.96
01-Nov-22	1400	47486	2.95
01-Dec-22	1405	47106	2.98
01-Jan-23	1396	46569	3.00
01-Feb-23	1374	45906	2.99
01-Mar-23	1412	45747	3.09
01-Apr-23	1393	45345	3.07
01-May-23	1378	45174	3.05
01-Jun-23	1397	45035	3.10
01-Jul-23	1401	44932	3.12
01-Aug-23	1398	44865	3.12
01-Sep-23	1386	44769	3.10
01-Oct-23	1407	44764	3.14
01-Nov-23	1384	44641	3.10
01-Dec-23	1462	44286	3.30
01-Jan-24	1449	44071	3.29
01-Feb-24	1435	43713	3.28
01-Mar-24	1412	43491	3.25
01-Apr-24	1395	43305	3.22
01-May-24	1391	43219	3.22
01-Jun-24	1418	42579	3.33
