# Supplementary material for: Prescribing of anti-dementia medications in primary care: A retrospective cohort study in 1489 English General Practices
Source: PLoS One. 2026 Jun 1;21(6):e0347921. doi: 10.1371/journal.pone.0347921 (PMC13225638; doi:10.1371/journal.pone.0347921)
Supplement: S7 Fig — (PDF) [file pone.0347921.s010.pdf]

Supplementary Table 3a: Accelerated failure time model with lognormal distribution (acetyl-cholinesterase inhibitor, n=242007)

	Coefficient (B)	Time ratio (e^B)	P	95%CI [lower]	95% CI [upper]
Gender					
**Female**	Ref				
**Male**	-0.03	**0.97**	0.08	-0.07	0.00
IMD Quintile					
**1 (least deprived)**	Ref				
**2**	0.15	**1.16**	**<0.01**	0.06	0.24
**3**	0.25	**1.28**	**<0.01**	0.15	0.36
**4**	0.42	**1.52**	**<0.01**	0.30	0.54
**5 (most deprived)**	0.52	**1.68**	**<0.01**	0.39	0.65
Ethnicity					
**Asian**	0.39	**1.48**	**<0.01**	0.24	0.54
**Black**	0.28	**1.32**	**<0.01**	0.12	0.44
**Mixed**	0.09	**1.09**	0.56	-0.22	0.40
**White**	ref		ref	ref	ref
**Other**	0.47	**1.6**	0.30	-0.42	1.35
**Unknown**	0.41	**1.51**	**<0.01**	0.20	0.62
**CKD diagnosis**					
**Yes**	-0.08	**0.92**	**<0.01**	-0.13	-0.03
**No**	Ref				
**Learning disability diagnosis**					
**Yes**	1.99	**7.32**	**<0.01**	1.73	2.26
**No**	Ref				
Region					
**London**	Ref				
**North East**	-0.57	**0.57**	**<0.01**	-0.87	-0.26
**North West**	0.13	**1.14**	0.31	-0.12	0.38
**Yorkshire and Humber**	0.03	**1.03**	0.84	-0.25	0.30
**East Midlands**	0.19	**1.21**	0.47	-0.32	0.69
**West Midlands**	0.52	**1.68**	**<0.01**	0.28	0.76
**East of England**	0.67	**1.95**	**<0.01**	0.37	0.98
**South West**	-0.13	**0.88**	0.27	-0.36	0.10
**South East**	0.14	**1.15**	0.23	-0.09	0.37
Age (at index date)	0.06	**1.06**	**<0.01**	0.06	0.07
Comorbidity Score	0.06	**1.06**	**<0.01**	0.05	0.07

Supplementary Table 3b: Accelerated failure time model with lognormal distribution (memantine, n=242007)					
	**Coefficient (B)**	**Time Ratio (e^B)**	**P**	**95%CI [lower]**	**95% CI [upper]**
**Gender**					
Female	Ref				
Male	-0.55	**0.58**	**<0.01**	-0.61	-0.50
**IMD Quintile**					
1 (least deprived)	Ref				
2	-0.03	0.97	0.7	-0.16	0.11
3	-0.02	0.98	0.76	-0.18	0.13
4	0.02	1.02	0.80	-0.15	0.19
5 (most deprived)	0.02	1.02	0.85	-0.17	0.20
**Ethnicity**					
**Asian**	0.26	**1.30**	**0.01**	0.07	0.45
Black	0.60	**1.82**	**<0.01**	0.38	0.83
Mixed	0.35	**1.42**	0.11	-0.08	0.78
White	ref				
Other	-0.08	**0.92**	0.87	-0.96	0.81
Unknown	0.29	**1.34**	0.24	-0.19	0.76
**CKD diagnosis**					
Yes	0.34	**1.40**	**<0.01**	0.26	0.42
No	Ref				
**LD diagnosis**					
**Yes**	1.37	**3.94**	**<0.01**	0.96	1.78
**No**	Ref				
**Region**					
London	Ref				
North East	-0.48	**0.62**	**0.01**	-0.85	-0.11
North West	-0.50	**0.61**	**<0.01**	-0.78	-0.22
Yorkshire and Humber	0.13	**1.14**	0.55	-0.29	0.55
East Midlands	0.78	**2.18**	**<0.01**	0.41	1.15
West Midlands	0.67	**1.95**	**<0.01**	0.35	0.99
East of England	1.39	**4.01**	**<0.01**	0.94	1.85
South West	-0.05	**0.95**	0.71	-0.31	0.21
South East	0.05	**1.05**	0.73	-0.22	0.32
**Age (at index date)**	0.02	**1.02**	**<0.01**	0.01	0.02
**Comorbidity Score**	0.01	**1.01**	0.148	0.00	0.02
