# Supplementary material for: Prescribing of anti-dementia medications in primary care: A retrospective cohort study in 1489 English General Practices
Source: PLoS One. 2026 Jun 1;21(6):e0347921. doi: 10.1371/journal.pone.0347921 (PMC13225638; doi:10.1371/journal.pone.0347921)
Supplement: S4 Table — (PDF) [file pone.0347921.s011.pdf]

**Supplementary Table 4a: Coefficients and Time Interaction Coefficients from flexible parametric models (AChEIs) (n=242,007)**					
	**Coefficient (B)**	**Time-interaction coefficients (γ)**	**P**	**95%CI [lower]**	**95% CI [upper]**
**Gender**					
Female	Ref	-			
Male	0.029	**-**	**<0.001**	0.014	0.043
**CKD diagnosis**					
Yes	0.061	**-**	**<0.001**	0.042	0.080
No	Ref	-			
**Learning disability diagnosis**					
Yes	-0.811	**-**	**<0.001**	-0.912	-0.711
No	Ref	-			
**Region**					
London	Ref	-			
North East	0.252	**-**	**<0.001**	0.142366	0.360791
North West	-0.027	-	0.549	-0.11581	0.061618
Yorkshire and Humber	0.034	-	0.486	-0.06085	0.127906
East Midlands	-0.058	-	0.514	-0.23276	0.116408
West Midlands	-0.137	**-**	**0.002**	-0.22106	-0.05208
East of England	-0.193	**-**	**0.001**	-0.30231	-0.0837
South West	0.058	-	0.172	-0.02528	0.141299
South East	0.005	-	0.901	-0.07502	0.085179
**Age (at index date)**	-0.02	**-**	**<0.001**	-0.025	-0.024
**Comorbidity Score**	-0.018	**-**	**<0.001**	-0.020	-0.016
**IMD Quintile***					
1 (least deprived)	Ref				
2	-	-0.011	**<0.001**	-0.016	-0.006
3	-	-0.018	**<0.001**	-0.023	-0.012
4	-	-0.025	**<0.001**	-0.031	-0.018
5 (most deprived)	-	-0.031	**<0.001**	-0.038	-0.024
**Ethnicity***					
White	Ref				
Asian	-	-0.025	**<0.001**	-0.032	-0.017
Black	-	-0.026	**<0.001**	-0.034	-0.018
Mixed	-	-0.008	0.366	-0.025	0.009
Other	-	-0.028	0.247	-0.074	0.019
Unknown	-	-0.025	**<0.001**	-0.035	-0.015

*Treated as time varying within the model

Interpretation of the time-interaction coefficients (γ): Supports the cox regression - those in more deprived IMDs, or who were black, Asian or Unknown ethnicity were less likely to receive the medication with the effect worsening over time.

Supplementary Table 4b: Coefficients and Time Interaction Coefficients from flexible parametric models (memantine) (n=242,007)					
	**Coefficient (B)**	**Time-interaction coefficients (γ)**	**P**	**95%CI [lower]**	**95% CI [upper]**
**Gender**					
Female	Ref	-			
Male	0.204	**-**	**<0.001**	0.185	0.223
**CKD diagnosis**					
Yes	-0.098	**-**	**<0.001**	-0.126	-0.070
No	Ref	-			
**Learning disability diagnosis**					
Yes	-0.546	**-**	**<0.001**	-0.694	-0.398
No	Ref	-			
**Region**					
London	Ref	-			
North East	0.177	**-**	**0.005**	0.054	0.301
North West	0.158	-	**0.001**	0.063	0.254
Yorkshire and Humber	-0.065	-	0.415	-0.220	0.091
East Midlands	-0.329	-	**<0.01**	-0.464	-0.194
West Midlands	-0.244	**-**	**<0.01**	-0.362	-0.126
East of England	-0.557	**-**	**<0.01**	-0.729	-0.384
South West	-0.004	-	0.926	-0.096	0.087
South East	-0.024	-	0.620	-0.120	0.072
**Age (at index date)**	-0.009	**-**	**<0.001**	-0.010	-0.008
**Comorbidity Score**	0.002	**-**	0.176	-0.001	0.006
**IMD Quintile***					
1 (least deprived)	Ref				
2	-	-0.004	0.219	-0.009	0.002
3	-	-0.006	0.084	-0.012	0.001
4	-	-0.008	**0.023**	-0.016	-0.001
5 (most deprived)	-	-0.012	**0.004**	-0.020	-0.004
**Ethnicity***					
White	-				
Asian	-	-0.015	**0.001**	-0.023	-0.006
Black	-	-0.030	**<0.001**	-0.040	-0.020
Mixed	-	-0.021	**0.045**	-0.042	0.000
Other	-	0.024	0.323	-0.024	0.072
Unknown	-	-0.012	0.235	-0.032	0.008

*Treated as time varying within the model

Interpretation of the time-interaction coefficients (γ): Again supports the cox model - less difference in chance of receiving IMD, seen only at the extremes. Asian, Black and mixed all significantly less likely to get memantine - with disparity growing over time.

*Treated as time varying within the model

Again supports the cox model - most dramatic differences with IMD, with more deprived quintiles significantly less likely to receive the medication. Asian, Black, Mixed and Unknown ethnicities, all significantly less likely to get memantine - with disparity growing over time.
