# Supplementary material for: Prescribing of anti-dementia medications in primary care: A retrospective cohort study in 1489 English General Practices
Source: PLoS One. 2026 Jun 1;21(6):e0347921. doi: 10.1371/journal.pone.0347921 (PMC13225638; doi:10.1371/journal.pone.0347921)
Supplement: Attachment — Submitted filename: Response_to_Reviewer_Comments_PLOS_V2.docx [file pone.0347921.s013.docx]

Division of Population Health, Health Services Research and Primary Care School of Health Sciences,

The University of Manchester,

Manchester,

UK

16^th^ March, 2026.

Dear Editor and Reviewers,

Thank you for taking the time to review our manuscript and provide the detailed feedback on the paper. The comments you have provided are helpful and strengthen the manuscript. Please see our detailed responses to your comments below. We hope these address your concerns regarding the paper.

---

1. Is the manuscript technically sound, and do the data support the conclusions?

**The manuscript must describe a technically sound piece of scientific research with data that supports the conclusions. Experiments must have been conducted rigorously, with appropriate controls, replication, and sample sizes. The conclusions must be drawn appropriately based on the data presented.**

**Reviewer #1: Partly**

**Reviewer #2: Yes**

Thank you for the comments. After the suggested revisions, we hope any concerns regarding the rigour of the research are addressed for reviewer 1.

**2. Has the statistical analysis been performed appropriately and rigorously?**

**Reviewer #1: Yes**

**Reviewer #2: Yes**

Thank you for the comments

**3. Have the authors made all data underlying the findings in their manuscript fully available?

The** [**PLOS Data policy [track.editorialmanager.com]**](https://urldefense.com/v3/__http:/track.editorialmanager.com/CL0/http:*2F*2Fwww.plosone.org*2Fstatic*2Fpolicies.action*23sharing/1/010f019cd05995af-c76cbd0d-7832-47cf-9e9e-22b4f737eb90-000000/vP-zIyPEU_wi9mLbnlSGtw4L6siJi1yMuPASS6uPwJw=253__;JSUlJSU!!PDiH4ENfjr2_Jw!B5W-aAG7xMxbkVPZfJIvoMVs84ZgX3T8uwJTqSWHPv_ZnIQNhDIv1VLmjVuN8Qx9hEuKJ9BTq--K7DKjuIuxsEfIFZXWpcRs$) **requires authors to make all data underlying the findings described in their manuscript fully available without restriction, with rare exception (please refer to the Data Availability Statement in the manuscript PDF file). The data should be provided as part of the manuscript or its supporting information, or deposited to a public repository. For example, in addition to summary statistics, the data points behind means, medians and variance measures should be available. If there are restrictions on publicly sharing data—e.g. participant privacy or use of data from a third party—those must be specified.**

**Reviewer #1: Yes**

**Reviewer #2: Yes**

Thank you for the comments

**4. Is the manuscript presented in an intelligible fashion and written in standard English?

PLOS ONE does not copyedit accepted manuscripts, so the language in submitted articles must be clear, correct, and unambiguous. Any typographical or grammatical errors should be corrected at revision, so please note any specific errors here.**

**Reviewer #1: Yes**

**Reviewer #2: Yes**

Thank you for the comments.

**5. Review Comments to the Author

Please use the space provided to explain your answers to the questions above. You may also include additional comments for the author, including concerns about dual publication, research ethics, or publication ethics. (Please upload your review as an attachment if it exceeds 20,000 characters)**

**Reviewer #1: This paper addresses some important questions about the prescribing of drugs for dementia in Primary Care and the impact of socioeconomic status and ethnicity.**

Thank you for agreeing about the importance of the work.

**However, I have the following comments:**
**Abstract Objective: does not mention cholinesterase inhibitors (AChEIs) specifically only memantine. As these drugs are a major interest of the study this should be corrected**

Thank you for pointing this out. It is important the abstract is clear, and it has been amended as suggested:

[Sentence added to: Abstract, OBJECTIVE]

*Patterns of prescribing of Acetyl Cholinesterase Inhibitors (AChEIs) and memantine require further exploration*

**Dementia Severity is a key difference in determining whether a person is started on treatment with an AChEI or memantine. The fact that the study was unable account for the effect of this on prescribing is a major limitation of the study. This is only mentioned in the discussion. This is an important issue that should receive more discussion and mention in methodology.**

We thank the reviewer for this insightful point. We agree that dementia severity is an important factor in prescribing decisions. We have expanded our discussion of this limitation and added clarifying text to the methods section to explain our approach.

In primary care electronic health records, the coding of even a dementia diagnosis is frequently suboptimal or absent^1-3^, although evidence is limited, coding of severity of dementia is likely even less accurate and more variable^4^. Restricting our cohort only to patients with a documented severity level at baseline would introduce significant selection bias and drastically reduce our sample size, potentially undermining the study’s power.

As dementia is a progressive condition, a mild baseline diagnosis often transitions to moderate or severe during the follow-up period. Furthermore, clinical guidelines evolved during our study period (as shown in Figure 1). This would complicate any analyses based on severity.

Under current and historical guidance (figure 1), the vast majority of patients with AD or LBD are eligible for either an AChEI or memantine at some stage of their illness. By including all patients regardless of documented severity, we capture the real-world clinical trajectory more holistically than a restricted documented severity subgroup would allow.

Sentences have been added in regarding this:

[Method: Classifying Dementia Subtype: Assessing Dementia Severity]

*Assessing Dementia Severity*

*We were unable to account for dementia severity at diagnosis due to the complexity of coding patterns for dementia within CPRD. There are codes such as ‘advanced dementia’ or ‘mild dementia’, however coding practices for even a diagnosis of dementia are variable and suboptimal^1-3^. Evidence is limited, but the accuracy and frequency of severity data is likely even worse^4^. Severity may also be recorded as free text which we were unable to access for analyses. Furthermore, any codes detailing severity may account for severity at diagnosis, but not how the condition progresses so if a mild dementia goes on to become moderate (as is clinically expected), the code may not change.*

[Discussion: Strengths and Limitations]

*This meant we could not determine eligibility for medications at baseline, or whether AChEIs or memantine should be offered. However, because dementia is a progressive condition a single baseline severity measure may not reflect eligibility for treatment over the entire study period. In clinical reality, most people with a diagnosis would be eligible for at least one of the examined medications at some point during the clinical trajectory (AChEI if diagnosed with mild or moderate; memantine if diagnosed when severe). This is an important limitation, and highlights the need for more detailed standardised dementia coding within primary care.*

**Tolerability of AChEIs: Memantine is indicated for people who are unable to tolerate AChEIs, and monotherapy with memantine would be appropriate for such patients; again it is not mentioned whether of not this could be determined from the CPRD database. This point is not considered in the discussion.**

Thank you for this helpful comment. You are entirely correct here with this point. Although codes suggesting medications were not tolerated are available, these would be inconsistently applied and limit the sample considerably and would introduce bias into analyses. Therefore, while we agree this is an important limitation, we also believe our methodology examining issues of prescriptions is reasonable and robust enough to answer our research questions. Nevertheless, a sentence recognising this important limitation has been added.

[Discussion: Strengths and Limitations].

*It was not possible to assess if patients were offered AChEIs or memantine and declined or did not tolerate the medications.*

**Coding Issues: apart from limitations of the CPRD database other coding issues are not discussed. It is well known that ethnicity is poorly coded in Primary care. This issue, how missing data was handled and the impact on the analysis and findings should be discussed.**
Thank you for the important point. Further sentences and important references have been added to the text to address this. By using the CPRD linked ethnicity data, we have used the best available data for ethnicity^5^. It was derived using CPRD’s algorithm which uses not only primary but also secondary care level data to provide an ethnicity value for each participant in the study. Previously, data for ethnicity were incomplete and poorly coded within primary care. However, the data for ethnicity recording within CPRD have improved in recent years and area not of high quality^5^. Missingness for ethnicity or IMD at the patient-level was very small for eligible patients (n=249, <0.04%); complete case analysis was conducted and given the small number of missing cases it is unlikely to have influenced results.

For other conditions, again missingness was low, because we deemed a code being absent as the condition not being present, not missing. We could have explored this further by looking at proxies for important diagnoses, e.g. issues of medications specific to each of the comorbidities included to increase accuracy. This was a limitation, which we have now discussed in the limitations section. However, as the comorbidity score was used as a covariate to adjust for chronic conditions, use of a code for the condition rather than detailed case finding is reasonable. We had wide ranging codelists, and considered a single instance of a code as the patient having the condition. All of the 19 comorbidities included are important medical conditions; if someone has one of these it is reasonable to assume that at some point a single code will have been entered into their primary care record.

[Methods: Demographics and comorbidities]

*If a condition was not coded, then it was deemed absent not missing.*

These represent the best available data for ethnicity within CPRD, and evidence suggests that CPRD ethnicity and IMD data is representative of the overall population and suitable for use in research^5, 6^

[Discussion: Strengths and Limitations]

*We took absence of a diagnostic code as absence of a condition in terms of the comorbidity score. Although this means we may have underestimated the comorbidity burden for some patients, as we had very wide ranging codelists and we used a single instance of the code as identifying the comorbidity, the number of patients affected is likely to be minimal*

**Other Factors: Two other things happened between 2006 and 2024: The DiADeM (Diagnosing Advanced Dementia Mandate) project, designed for use in care homes in England, was launched as a national pilot in 14 sites by NHS England in 2022. Some of these sites were included in the areas included in this study. Memantine would be more likely to be used in people with advanced dementia. The implications of this need to be considered as a reason for monotherapy prescriptions in the data**

**COVID: the impact of Covid delaying diagnosis and on initiating prescribing of AChEIs and / or memantine should be discussed. During Covid there was an upsurge in the prescription of antipsychotic drugs for behavioural issues – were these used instead of trial of memantine?**

Thank you for raising these important points. We have addressed them by referencing the time series analyses which did not show dramatic shifts around the time of the covid pandemic, or when drives for diagnosis were being conducted by NHS England.

[Discussion: Results in the context of other literature]

*The study covers the period of the Covid-19 pandemic (2020-22), however the time series analyses did not show any dramatic changes in prescribing levels over these months. This may be because prescribing continued, or because there were fewer diagnoses and fewer prescriptions issued, meaning overall percentages remained the same. Other large important studies going on during this period included NHS England drives to diagnose dementia^7, 8^ and changes to guidance as shown in figure 1Despite thiswe did not see notable shifts in monthly prescribing levels that corresponded with these events.*

**---**

**Reviewer #2: This is. very important paper that demonstrates a lack of prescribing and inequality of prescribing effective, safe & cheap medications for several types of dementia.**
Thank you for the comment about the importance of the study, with which we fully agree.

**I would suggest the following edits**

**In the introduction I suggest adding this meta analysis as the efficacy of ACEI is higher in DLB than AD and this may not be well known and may reduce prescribing in that condition**
 **Knight R, Khondoker M, Magill N, Stewart R, Landau S. A Systematic Review and Meta-Analysis of the Effectiveness of Acetylcholinesterase Inhibitors and Memantine in Treating the Cognitive Symptoms of Dementia. Dement Geriatr Cogn Disord. 2018;45(3-4):131-151. doi: 10.1159/000486546. Epub 2018 May 7. PMID: 29734182.**

**This may explain why prescribing for ACHEI is higher in DLB. This is good but still a large percentage of eligible patients are not being prescribed**
Thank you for pointing out this important and interesting reference. It is highly relevant and also important to highlight this to readers to increase awareness. A sentence has been added and the reference.

[Introduction]

*Systematic-review evidence suggests that the efficacy of AChEIs is actually higher in those with LBD than AD^9^, but this may not be well known particularly among non-specialists within primary care, and lead to lower rates of prescribing in this subtype.*

**In the section ‘Implications for practice policy and research ‘
There is a sentence, ‘New curative drugs for AD33, 34 are currently unavailable, due to a lack of cost-effectiveness data, therefore we should be optimising prescribing of existing drugs to all who can benefit. ‘
Please change to
New disease modifying anti-amyloid drugs for AD33, 34 are currently unavailable in the NHS, due to a lack of cost-effectiveness data, therefore we should be optimising prescribing of existing drugs to all who can benefit. ‘**

**They do not cure AD. There is no cure. The drugs are available, but you have to pay for them. Approximate cost is £70K to include prescribing drugs and MRI monitoring**

Thank you for this detailed comment and your attention to detail here. The sentence has been changed as suggested.

[Implications for practice, policy and research]

*New disease modifying anti-amyloid drugs^10, 11^ are currently unavailable on the NHS, due to a lack of cost-effectiveness data, therefore we should be optimising prescribing of existing drugs to all who can benefit*

**Could the authors comment or add in that these drugs are very cheap (see below)
COST of Donepezil
• Standard Tablet Cost: According to the NHS Drug Tariff (Part VIIIA Category M), the basic price for 28 tablets of donepezil (both 5mg and 10mg) is often in the region of £0.94 to £1.04.
• Oral Solution: 150ml of 1mg/ml sugar-free oral solution is priced at approximately £118.63.
Based on the NHS Electronic Drug Tariff and formulary, the costs for rivastigmine vary by formulation (capsules or patches) and strength. As of recent data, generic rivastigmine is generally cost-effective, but specific formulations can differ:
• Capsules (28-pack, various strengths): Prices generally range from £1.70 to £2.40 for 28 capsules, depending on the manufacturer.
• Transdermal Patches (13.3mg/24hours, 30 pack): The basic price is approximately £77.97.
• Oral Solution (2mg/ml, 120ml): The basic price is approximately £96.82 - £99.14.
• Orodispersible Tablets: These are very expensive, with 10mg tablets costing roughly 5 times more than 5mg tablets.
Based on the NHS Drug Tariff and BNF (British National Formulary), the cost of memantine varies depending on the form, strength, and whether the branded version (Ebixa) or generic is prescribed. Generic memantine is significantly cheaper for the NHS.
Approximate NHS Costs (Drug Tariff Part VIIIA, 2026):
• Memantine 10mg Tablets (Pack of 28): Roughly £1.24.
• Memantine 20mg Tablets (Pack of 28): Roughly £1.91.
• Memantine 10mg/ml Oral Solution (50ml): Approximately £8.94.
• Orodispersible Tablets (Sugar-free, 28 tablets): These are much more expensive, often costing between £12.50 (5mg) and £49.98 (20mg)**

Thank you for the detailed comment, with which we absolutely agree. A further sentence has been added about how these are cheap effective medications. We have also pointed out they are available in different forms which is a contrast to the new AD anti-amyloid therapies.

[Discussion: Implications for practice, policy and research]

*AChEIs and memantine are very cheap, available in multiple forms, with evidenced benefits to cognition, and their potential benefit to all eligible patients need to be optimised.*

**Could the authors add anything about safety. Can they see how many people were prescribed these drugs and could not tolerate them?**

Thank you for raising this point, which echoes that of reviewer 1. We have described above the complexity around this, and added in a sentence to the limitations section.

*We did not assess if patients were offered AChEIs or memantine and declined or did not tolerate the medications. This was due to the, accuracy and variability of how this was coded limiting inference.*

**In the implication section can the authors include that Annual reviews by GPs are a key part of a person with dementia's care.
Annual dementia reviews are funded by the NHS as part of primary care (general practice) services, specifically under the Quality and Outcomes Framework (QOF) in England. They do not need to be undertaken by a GP but should bestructured annual review with a GP or nurse to monitor symptoms, medication, and support needs**

**Could the implication comment on are these annual reviews monitored. Could medication revie be included. Could pharmacists flag up cases where there is a demenatoi diagnosis and where there are no ACHEI or memantine prescribed?**

Thank you for raising this important point, highly relevant to primary care. Further information has been included about this as detailed below. We absolutely agree that medication should be considered within the annual review by whichever clinician is conducting it. We have also included recent studies that evidence that high rates of reviews are recorded, but quality is variable, particularly perceived by people with dementia in areas of socio-economic deprivation.

[Discussion: Implications for practice, policy and research]

*Annual reviews and care plans within primary care are a key part of healthcare for people with dementia^12^. These are financially incentivised by the Quality and Outcomes Framework (QOF)^13^, and guidance suggests these would include a review of medications, including eligibility for anti-dementia medications^12^. Evidence suggests that these reviews are happening at high rates across subtypes^14^, but that quality is variable and patients are not always aware these have taken place^15, 16^. Clinicians conducting these annual reviews should be aware of these findings, and for those with AD or LBD dementias ensure that appropriate anti-dementia therapy has been considered*.

We look forward to your reply

Kind regards

Dr Charlotte Morris

Corresponding author

References:

1. Ford E, Greenslade N, Paudyal P, Bremner S, Smith HE, Banerjee S, et al. Predicting dementia from primary care records: A systematic review and meta-analysis. PLoS ONE. 2018;13(3):e0193732.

2. Russell P, Banerjee S, Watt J, Adleman R, Agoe B, Burnie N, et al. Improving the identification of people with dementia in primary care: evaluation of the impact of primary care dementia coding guidance on identified prevalence. BMJ Open. 2013;3(12):e004023.

3. Wilkinson T, Schnier C, Bush K, Rannikmäe K, Henshall DE, Lerpiniere C, et al. Identifying dementia outcomes in UK Biobank: a validation study of primary care, hospital admissions and mortality data. Eur J Epidemiol. 2019;34(6):557-65.

4. Prakash R, Dupre ME, Østbye T, Xu H. Extracting Critical Information from Unstructured Clinicians' Notes Data to Identify Dementia Severity Using a Rule-Based Approach: Feasibility Study. JMIR Aging. 2024;7:e57926.

5. Shiekh SI, Harley M, Ghosh RE, Ashworth M, Myles P, Booth HP, et al. Completeness, agreement, and representativeness of ethnicity recording in the United Kingdom’s Clinical Practice Research Datalink (CPRD) and linked Hospital Episode Statistics (HES). Popul Health Metr. 2023;21(1):3.

6. Mahadevan P, Harley M, Fordyce S, Hodgson S, Ghosh R, Myles P, et al. Completeness and representativeness of small area socioeconomic data linked with the UK Clinical Practice Research Datalink (CPRD). J Epidemiol Community Health. 2022;76(10):880.

7. NHS England. NHS launches new dementia diagnosis drive [Internet]. 2022 [cited 2026 Mar 16]. Available from: [https://www.england.nhs.uk/2022/05/nhs-launches-new-dementia-diagnosis-drive/](https://www.google.com/search?q=https://www.england.nhs.uk/2022/05/nhs-launches-new-dementia-diagnosis-drive/)

8. Department of Health. Prime Minister's challenge on dementia 2020 [Internet]. London; 2015 [cited 2026 Mar 16]. Available from: <https://www.gov.uk/government/publications/prime-ministers-challenge-on-dementia-2020>

9. Knight R, Khondoker M, Magill N, Stewart R, Landau S. A Systematic Review and Meta-Analysis of the Effectiveness of Acetylcholinesterase Inhibitors and Memantine in Treating the Cognitive Symptoms of Dementia. Dement Geriatr Cogn Disord. 2018;45(3-4):131-51.

10. Frisoni GB, Aho E, Brayne C, Ciccarelli O, Dubois B, Fox NC, et al. Alzheimer's disease outlook: controversies and future directions. The Lancet. 2025;406(10510):1424-42.

11. Fox NC, Belder C, Ballard C, Kales HC, Mummery C, Caramelli P, et al. Treatment for Alzheimer's disease. The Lancet. 2025;406(10510):1408-23.

12. National Institute for Health and Care Excellence. Dementia: assessment, management and support for people living with dementia and their carers [Internet]. NICE guideline NG97; 2018 [cited 2026 Mar 16]. Available from: <https://www.nice.org.uk/guidance/ng97>

13. NHS England. Quality and outcomes framework guidance for 2022/23 [Internet]. 2022 [cited 2026 Mar 16]. Available from: [https://www.england.nhs.uk/publication/quality-and-outcomes-framework-guidance-for-2022-23/](https://www.google.com/search?q=https://www.england.nhs.uk/publication/quality-and-outcomes-framework-guidance-for-2022-23/)

14. Morris CM, Pearl, Robinson L, Ashcroft D, Blakeman T, Kontopantelis E. How does diagnostic subtype affect the quality of primary care for people with dementia? A retrospective cohort study in 1490 English General Practices. Age Ageing. 2026; In Press.

15. Connolly A, Iliffe S, Gaehl E, Campbell S, Drake R, Morris J, et al. Quality of care provided to people with dementia: Utilisation and quality of the annual dementia review in general practice. Br J Gen Pract. 2012;62(595):e91-8.

16. Morris C, Robinson L, Ashcroft D, Kontopantelis E, Blakeman T. Experiences of primary care for people with dementia from socio-economically disadvantaged areas: a qualitative study. Br J Gen Pract. 2026;76(764).
